# Patient-reported outcome measures in physical therapy practice for neck pain: an overview of reviews

**DOI:** 10.1186/s41687-023-00637-0

**Published:** 2023-10-02

**Authors:** Michelle M. Ramirez, Mark H. Shepherd, S. Jacob Melnick, Cannon Hanebuth, Caroline Bazemore, Logan Couce, Steph Hendren, Maggie E. Horn

**Affiliations:** 1 Department of Population Health Sciences, Department of Orthopaedic Surgery, Duke University School of Medicine, 215 Morris Street, Suite 200, Durham, NC 27708 USA; 2 Department of Physical Therapy, Bellin College, 3201 Eaton Rd, Greenbay, WI 54311 USA; 3 Doctor of Physical Therapy Program, Hawai’i Pacific University, 500 Ala Moana Blvd, Honolulu, HI 96813 USA; 4 Division of Physical Therapy, Duke University School of Medicine, DUMC Box 104002, Durham, NC 27710 USA; 5 Sugar House Health Center, University of Utah, 1280 E. Stringham Ave, Salt Lake City, UT 84106 USA; 6 Research & Education Librarian, Duke University Medical Center Library & Archives, Seeley G. Mudd Bldg., 103, Durham, NC 27710 USA; 7 Department of Orthopaedic Surgery, Division of Physical Therapy, Department of Population Health Sciences, Duke University School of Medicine, DUMC Box 104002, Durham, NC 27710 USA

**Keywords:** Neck pain, Outcomes, Patient-reported outcome measures

## Abstract

**Background:**

Understanding which patient-reported outcome measures are being collected and utilized in clinical practice and research for patients with neck pain will help to inform recommendations for a core set of measures that provide value to patients and clinicians during diagnosis, clinical decision-making, goal setting and evaluation of responsiveness to treatment. Therefore, the aim of this study was to conduct a review of systematic reviews using a qualitative synthesis on the use of patient-reported outcome measures (PROMs) for patients presenting with neck pain to physical therapy.

**Methods:**

An electronic search of systematic reviews and guideline publications was performed using MEDLINE (OVID), Embase (Elsevier), CINAHL Complete (EBSCOhost), and Web of Science (Clarivate) databases to identify reviews that evaluated physical therapy interventions or interventions commonly performed by a physical therapist for individuals with neck pain and included at least one patient-reported outcome measure. The frequency and variability in which the outcome measures were reported among the studies in the review and the constructs for which they measured were evaluated. The evaluation of a core set of outcome measures was assessed. Risk of bias and quality assessment was performed using A Measurement Tool to Assess systematic Reviews 2.

**Results:**

Of the initial 7,003 articles, a total of 37 studies were included in the final review. Thirty-one PROMs were represented within the 37 reviews with eleven patient-reported outcome measures in three or more reviews. The eleven PROMs assessed the constructs of disability, pain intensity, psychosocial factors and quality of life. The greatest variability was found amongst individual measures assessing psychosocial factors. Assessment of psychosocial factors was the least represented construct in the included studies. Overall, the most frequently utilized patient reported outcome measures were the Neck Disability Index, Visual Analog Scale, and Numeric Pain Rating Scale. The most frequently used measures evaluating the constructs of disability, pain intensity, quality of life and psychosocial functioning included the Neck Disability Index, Visual Analog Scale, Short-Form-36 health survey and Fear Avoidance Belief Questionnaire respectively. Overall risk of bias and quality assessment confidence levels ranged from critically low (2 studies), low (12 studies), moderate (8 studies), and high (15 studies).

**Conclusion:**

This study identified a core set of patient-reported outcome measures that represented the constructs of disability, pain intensity and quality of life. This review recommends the collection and use of the Neck Disability Index and the Numeric Pain Rating Scale or Visual Analog Scale. Recommendation for a QoL measure needs to be considered in the context of available resources and administrative burden. Further research is needed to confidently recommend a QoL and psychosocial measure for patients presenting with neck pain. Other measures that were not included in this review but should be further evaluated for patients with neck pain are the Patient Reported Outcomes Measurement Information System (PROMIS) Physical function, PROMIS Pain Interference and the Optimal Screening for Prediction of Referral and Outcome Yellow Flag (OSPRO-YF) tool.

**Supplementary Information:**

The online version contains supplementary material available at 10.1186/s41687-023-00637-0.

## Background

It is well understood that “measuring health is the first step to understanding health and understanding health is the first step to improving health” [1]. Patient-reported outcome measures (PROMs) present the opportunity to capture information directly from the patient that can help clinicians and researchers understand the impact of disease, treatment, and health status directly as the patient perceives it [2]. Moreover, PROMs play a critical role in supporting shared decision-making and personalized goal-setting between a patient and provider [2, 3]. In high-burden diseases with multifactorial causes, such as neck pain, PROMs present an opportunity to capture information that can inform the development of individualized evidence-based interventions, assess responsiveness to treatment and inform prognosis beyond traditional objective assessments [4].

Various evidence-based interventions have been recommended for the treatment of neck pain including treatments provided by a variety of interdisciplinary clinicians. However, due to the lack of standardization of PROMs across disciplines and in many cases even within a single discipline, there is difficulty in comparing the outcomes of these interventions [5]. This heterogeneity of measures makes it challenging to quantitatively evaluate which treatments are effective, their use in clinically meaningful research and comparison of findings between studies [3]. To that end, the expansion of electronic health record capabilities and data management allow the aggregation of large scale data collection and patient reported outcome integration at an unprecedented level. However, with the continued heterogeneity of measurement use in patients with neck pain and without minimal mandates, the ability to use this data to improve patient outcomes and advance the field will remain suboptimal.

Standardized PROM use has the potential to complement a clinician’s experience and expertise with an objective assessment of a patient’s status as they perceive it, assist with shared decision making, detect improvement in function, and provide informative large scale data to drive value based care pathways and quality improvement [6]. Various professional organizations, including the American Physical Therapy Association(APTA) have included recommendations for standardized PROM collection within published clinical practice guidelines(CPGs) including those specific to neck pain [4]. Despite open access to these guidelines, continued inconsistencies and lack of standardization in PROMs exist. To that end, these inconsistencies subsequently reduce the value of PROMs within physical therapy and across other professions [7].

Continued challenges to their implementation into clinical practice has been attributed to multifaceted barriers including lack of time to complete questionnaires, administrative burden, and lack of knowledge on how to translate data to knowledge [7, 8]. Additionally, without standardization of PROMs, patients may face “survey fatigue”. This combined with a clinician’s potential lack of knowledge on how to use the results to inform their clinical decision making further enhances the patients’ assumptions that they provide little value to their care. To that end, it’s critical to consider a PROMs measurement characteristics such as validity, consistency, feasibility, interpretability, and responsiveness. Therefore, a thoughtful, pragmatic, and evidence-informed selection process will ultimately influence the extent that the measure will be valuable, useful, and informative in clinical practice [1, 9].

In 2019, Chiarotto described three-steps to guide selection of the most appropriate PROM for a particular context [10]. Understanding what you want to measure and for what purpose, reviewing the literature, and assessing the quality of the measurement tool of interest were recommended steps to ensure what matters most to patients is captured [10]. Additionally, utilization of a conceptual model and framework to guide appropriate patient-reported outcome selection has also been suggested [11]. Physical therapists are one of the primary non-operative providers for patients with neck pain [12]. Accordingly, patients with neck pain account for approximately 20% of patients referred to outpatient physical therapy [13]. The first step to making recommendations for a set of PROMs to be used for patients with neck pain is to understand the breadth of PROMs within the profession. Secondly, it’s critical to understand what patient populations and clinical context these PROMs are reported in the literature. Therefore, the purpose of this study was to identify PROMs that are reported in patients with neck pain receiving physical therapy interventions and to provide guidance for physical therapists and other practitioners on PROM selection in this patient population.

## Methods

### Review design

The protocol for this systematic review was designed in accordance with the Preferred Reporting Items for Systematic Reviews and Meta-analysis (PRISMA) guidelines [14] and is registered with the International Prospective Register of Systematic Reviews (PROSPERO) database (CRD42023391158) [15]. We collaborated with a research librarian (SH) to develop an appropriate search strategy and management of the literature review.

### Data sources and search strategy

We searched MEDLINE (OVID), Embase (Elsevier), CINAHL Complete (EBSCOhost), and Web of Science (Clarivate) on September 13, 2022, using a combination of keywords and database-specific subject headings for the following concepts: neck pain, including any conditions that had a primary symptom of pain, and specific outcomes identified of interest by the group. An additional modified filter from the COnsensus-based Standards for the selection of health status Measurement Instruments (COSMIN) was used to further limit studies that mentioned reliability and validity information [16]. No restrictions were placed by date or language. The search was limited to only systematic review and guideline publications using two Canadian Agency for Drugs and Technologies in Health (CADTH) search hedges, which were only modified to remove the health technology assessment terms. The search strategies were peer-reviewed by another librarian with expertise in systematic review searches prior to execution using the Peer Review of Electronic Search Strategies (PRESS) checklist [17]. The full, reproducible search strategies for all included databases are available in supplementary material 1.

### Inclusion and exclusion criteria

The inclusion criteria for this study were systematic reviews of patients of any age or sex with neck pain receiving a physical therapy intervention or interventions commonly performed by a physical therapist. Studies included in this review must have met the additional criteria of reported outcomes in patients 18 years or older, patients with neck pain or cervicogenic headaches, and at least one patient-reported outcome measure recorded. The exclusion criteria applied in this study were if the study design was anything other than a systematic review of studies that used an experimental, quasi-experimental, or observational design, patients evaluated had neck pain with another spine-related condition such as low back pain, the intervention was provided by a chiropractor or the patient population included patients with neck pain who had neurologic deficits, severe cardiovascular diseases, serious pathology (e.g., malignancy, infection, cancer, inflammatory arthritis, fractures, upper cervical instability, etc.).

### Study selection and data extraction

After databases were searched, titles and abstracts of studies were uploaded into Covidence. The article selection process was completed in two phases. In the first phase, two authors (MR and MH) performed independent reviews of titles and abstracts in Covidence using the predefined inclusion and exclusion criteria above. Articles were moved to full-text review if one or both authors found the article potentially relevant. In the second phase, the same two authors independently reviewed full-text articles for eligibility. Any conflicts were resolved by discussion between authors. Three reviewers (MR, MH, MS) performed independent data extraction with a checked final review performed by a single reviewer (MR). Data extraction was performed using a Population, Intervention, Comparison, Outcome (PICO) format with elements representing author, year, title, databases searched, study type, number of included studies, population, intervention, comparators, and patient-reported outcome measures evaluated. Any other measures included in the reviews were also extracted.

### Data analysis and synthesis

Studies included in this review were evaluated from December 2022 to February 2023. The primary purpose of this review was to describe PROMs in physical therapy research and practice through qualitative synthesis. Therefore, we did not perform a meta-analysis of the data. For the qualitative synthesis, we described the studies by publication year, clinical population, study type, number of studies included in the review, and the outcomes reported in each study. We reported the frequency of PROMs by the constructs of disability, pain intensity, psychosocial factors, and quality of life. These were described according to their context of use (diagnosis, prognosis, and/or risk) within the included reviews.

### Risk of bias

Two review authors (CH and JM) independently assessed the risk of bias in included reviews using the Assessment of Multiple Systematic Reviews 2 (AMSTAR 2) tools. AMSTAR 2 is a validated instrument that uses 16 questions to assess systematic reviews that include randomized and non-randomized studies of healthcare interventions, or both [18]. The included studies were appraised according to AMSTAR 2 guidance and rated the reviews into four categories: “high”, “moderate”, “low”, and “critically low” in overall confidence. We considered the potential impact of an inadequate rating for each item individually. Particularly, we took into account the critical domains, which include whether or not a protocol was registered before the commencement of the review, the adequacy of the literature search, the justification for excluding individual studies, the risk of bias from individual studies being included in the review, consideration of the risk of bias when interpreting the results of the review and the assessment of the presence and likely impact of publication bias. Disagreements between the review authors over the risk of bias in particular studies were resolved by consensus.

## Results

### Study characteristics

**Fig. 1:**
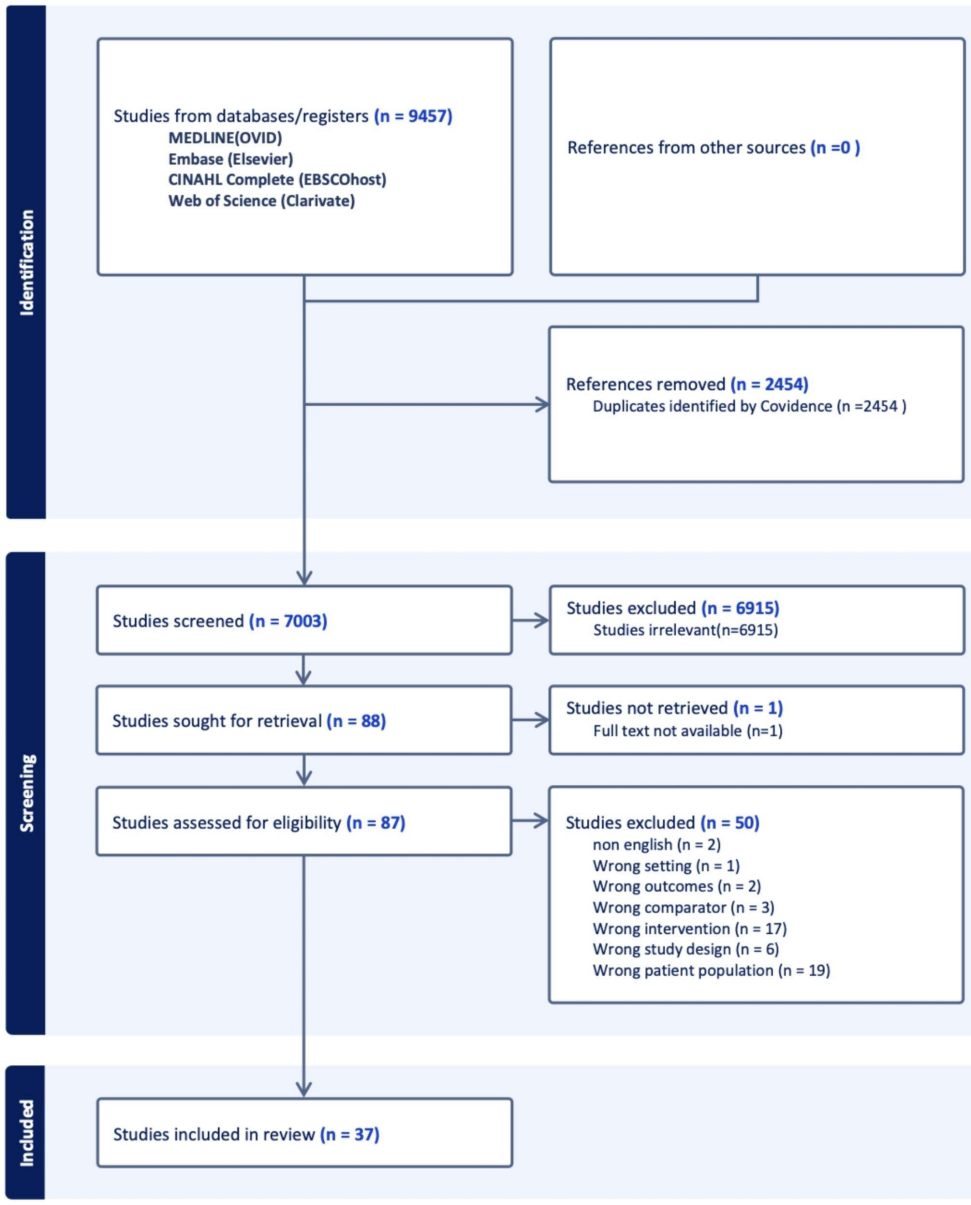
PRISMA flow diagram of screened and eligible citations

The electronic search resulted in an initial 9457 articles (Fig. 1). After 2454 duplicates were removed, 7003 articles were included for abstract and title review. Eighty-eight articles met the inclusion/exclusion criteria and were included in full-text retrieval. One study was excluded due to lack of full-text availability. After a full-text review, a total of 50 studies were excluded. This was due to the wrong patient population (19), wrong intervention (17), wrong study design (6), wrong comparator (3), wrong outcomes (2), non-English (2), and wrong setting (1).

Thirty-seven studies were included in the final review, were published between 2015 and 2022 and included a total of 31 distinct PROMs reported across all studies. Detailed characteristics of the included studies and PROMs are described and summarized in Table 1. Of the studies extracted for final review, 17 were systematic reviews and 20 were systematic reviews with meta-analyses. The mean number of studies included within each review was 13 (range 4–51). 70% of reviews included individuals with non-specific neck pain (acute, sub-acute, chronic), 27% included study populations specifically with whiplash-associated disorder (WAD), 27% included systematic reviews of individuals with radiating pain (radicular), and 22% of studies included populations consistent with cervicogenic headache. There were fourteen studies that included more than one study population within their review. There were a total of thirty-one PROMs reported across the thirty-seven studies in patients with non-specific neck pain, WAD, radiating pain and cervicogenic headache. Four patient-reported outcome constructs were identified amongst the included measures (Table 2). This included the constructs of disability, pain intensity, psychosocial factors, and QoL.

**Table 1:** Description and characteristics of included reviews

Study	Databases Searched	Study Type	Studies, n	Clinical population	Patient reported outcomes
Amiri A et al. (2017) [33]	Cochrane Library Google Scholar OVID PEDro PubMed ScienceDirect	SR of RCT’s	9	NS Neck Pain	NDI VAS
Araujo et al. (2017) [34]	CINAHL Embase MEDLINE PsycINFO Scopus Web of Science	SR with MA	7	NS NP with or without radicular symptoms, NP with headache	NDI NPAD NPQ VAS
Borrella-Andrés et al. (2021) [21]	Cochrane Library Plus PEDro PubMed Scopus Web of Science	SR of RCT’s	17	NP with radiating pain	NDI NPRS NPQ PSFS SF-36 SF-MPQ VAS
Chaibi A et al(2021) [54]	CENTRAL CINAHL EMBASE MEDLINE OpenGrey Ovid Web of Science	SR with MA	6	Acute neck pain	NPRS VAS
Cox L et al. (2019) [12]	CINAHL EMBASE EMBASE Classic MEDLINE PEDro PsycINFO	SR	5	Chronic NS NP, NP with WAD	NDI PDI
Dorji K et al. (2022) [35]	AMED CINAHL EMBASE MEDLINE PEDro PubMed	SR of RCT’s	6	NS Neck Pain	NDI NPDS NPRS
Fernandez M et al. (2020) [32]	Cochrane Central Register of Controlled Trials Mantis MEDLINE PEDro	SR with MA	7	NP with headache	HIT-6 MVKS NDI NPRS VAS
Fredin K. et al. (2017) [22]	AMED (Ovid) CENTRAL EMBASE (Ovid) MEDLINE (Ovid) PEDro	SR with MA	7	NS Neck Pain	NDI NPRS NPQ SF-12 SF-36 VAS
Garzonio S et al. (2022) [23]	CINAHL Embase MEDLINE PEDro The Cochrane Library	SR with MA	25	NS neck pain, Neck Pain with WAD	VAS NPRS
Gross A et al. (2015) [40]	CENTRAL CINAHL EMBASE MEDLINE	SR	51	NS Neck Pain, NP with radiating pain, NP with headache	NDI NPRS SF-36
Hanel J et al. (2020) [41]	CENTRAL CINAHL EMBASE MEDLINE SPORTDiscus	SR with MA	30	Chronic neck pain	FABQ TSK
Lantz JM et al. (2021) [36]	CENTRAL EMBASE PEDro PubMed Web of Science	SR	6	Post-op neck pain	EQ-5D NDI VAS
Liang et al. (2019) [24]	Chinese National Knowledge Infrastructure Database EMBASE PubMed Cochrane Library VIP database Wanfang database Web of Science	SR with MA	10	NP with radiating pain	NDI SF-12 SF-36 VAS
Lin et al. (2021) [25]	Airiti Library China National Knowledge Infrastructure CINAHL (via EBSCO) EMBASE (via Elsevier) PEDro ProQuest PubMed The Cochrane Central Register of Controlled Trials (via Wiley Online Library) Wanfang Data	SR with MA	11	NS NP, NP with radiating pain, Neck pain with WAD, NP with headache	FABQ HSCL NDI NNP NPQ NPRS SF-36 VAS
Louw S et al. (2017) [26]	BioMed Central CINAHL Cochrane library PEDro PubMed ScienceDirect Scopus	SR with MA	8	NS Neck Pain	DASH NDI NPQ NPRS SF-36 VAS
Mallard F et al.(2022) [42]	APA PsycInfo CINAHL EMBASE Index to Chiropractic Literature MEDLINE PEDro PubMed SportDiscus the Cochrane Central Register of Controlled Trials	SR	4	NP with radiating pain	NDI NPRS
Martimbianco A et al. (2019) [43]	CENTRAL CINAHL Clinicaltrials.gov CRS EMBASE ICTRP LILACS MEDLINE OPENSIGLE PEDro PubMed	SR	7	chronic neck pain, neck pain with WAD, NP with headache	NDI SF-36 VAS
Martin-Gomez C. et al. (2019) [44]	Cochrane PEDro PubMed Scopus Web of Science	SR with MA	10	Chronic neck pain	NDI NPRS VAS
Masaracchio M et al. (2019) [45]	AMED CINAHL Clinicaltrials.gov Cochrane Library EMBASE PEDro PubMed	SR with MA	14	Mechanical neck pain	NDI NPQ NPRS VAS
Monticone M. et al. (2015) [27]	CENTRAL CINAHL ClinicalTrials.gov EMBASE MEDLINE PsycINFO PubMed Scopus Web of Science World Health Organization International Clinical Trials Registry Platform	SR	10	Subacute and chronic NP	FABQ NDI NPRS SF-36 TSK
Nunez-Cabaleiro et al. (2022) [28]	CINAHL MEDLINE PEDro PubMed Scopus Web of Science	SR	14	NP with headaches	HI NDI NPRS
Price J et al. (2020) [46]	CINAHL EMBASE MEDLINE PEDro	SR	26	Chronic NS neck pain; Neck pain with WAD, NP with headaches	ADLQ DASH NDI NPQ PSFS VAS
Qing W. et al. (2021) [37]	CENTRAL (via The Cochrane Library) EMBASE (via Ovid) PEDro PubMed	SR with MA	12	Mechanical neck pain	NDI NHP NPRS
Rampazo E. et al. (2022) [47]	CENTRAL (via The Cochrane Library) EMBASE (via Ovid) PEDro PubMed	SR	30	NS neck pain	Becks Depression Inventory Goldeberg scale NDI NPAD NPQ SF-12 SF-36 VAS
Rodriguez-Huguet et al. (2022) [39]	Cochrane Library PEDro PubMed Scopus Web of Science	SR	11	Chronic neck pain	NDI NPQ NPRS VAS
Romeo A. et al. (2018) [29]	CINAHL COCHRANE Controlled Trials Register ISI Web of Science PEDro PubMed Scopus	SR with MA	5	NP with radiating pain	FABQ NDI NPRS PSFS VAS
Southerst D. et al. (2016) [38]	CINAHL EMBASE Index to Chiropractic Literature MEDLINE PsycINFO PubMed the Cochrane Central Register of Controlled Trials the Database of Abstracts of Reviews of Effects	SR	11	Neck pain and WAD	CES-D GSE NDI NPAD NPQ NPRS POMS SF-36
Tsiringakis G et al. (2020) [48]	COCHRANE EBSCOhost EMBASE Google Scholar MEDLINE PEDro PubMED SportDiscus	SR with MA	17	NS neck pain	NDI NPRS VAS
Varangot-Reille et al. (2022) [49]	CINAHL Google Scholar MEDLINE (PubMed) PEDro	SR with MA	22	NS neck pain, NP with radiculopathy, NP with headache	DASH FABQ NDI MPQ NPQ NPRS PSFS QDASH VAS
Villanueva-Ruiz I et al. (2022) [30]	MEDLINE (PubMed) PEDro Scopus Web of Science	SR with MA	12	Chronic NS neck pain	NDI NPRS VAS
Visvanathan R et al. (2018) [19]	ACRM American Physical Therapy Association BIOMED CENTRAL EMBASE Europa Medicophysica BMJ Medicine and Science in sports and exercise MEDLINE PEDro	SR	23	Chronic neck pain, NS neck pain, NP with WAD	HQoLQ NDI NPRS VAS VNDI
Wang S et al. (2022) [50]	Cochrane Library EMBASE PubMed Web of Science	SR with MA	12	Subacute neck pain, NP with radiating pain	VAS NPQ NPRS
Wilhelm M et al. (2020) [51]	CINAHL Cochrane Central Register of Controlled Trials PEDro SPORTDiscus PubMed Scopus	SR with MA	14	Acute, subacute, or chronic neck pain	NDI NPAD NPRS VAS
Wu. et al. (2020) [83]	Cochrane Library EBSCO Information Services EMBASE Web of Science	SR with MA	6	NS neck pain	NDI
Yang J et al. (2017) [31]	Cochrane Library Embase PubMed Scopus	SR with MA	7	NS neck pain, NP with radiating pain	MPQ NDI NPRS PSFS VAS
Yu H. et al. (2016) [52]	CINAHL Cochrane Central Register of Controlled Trials Database of Abstracts of Reviews of Effects (DARE) EMBASE Index to Chiropractic Literature (ICL) MEDLINE PsycINFO PubMed	SR	6	NS neck pain, NP with WAD	GHQ-28 (emotional distress) IES NDI NPQ NPRS PSFS SF-12 SF-36 TSK VAS
Zacharakis A et al. (2020) [53]	Cochrane Library EMBASE MEDLINE Scopus	SR of RCT’s	5	Acute neck pain, Chronic NP	ADLQ NDI NPRS VAS

Abbreviations: ADLQ, activities of daily living questionnaire; BDI, becks depression inventory; CES-D, center for epidemiologic studies depression scale; DASH, disabilities of the arm, shoulder and hand; EQ-5D,EuroQoL-5D; FABQ, fear avoidance belief questionnaire; GSES, general self-efficacy scale; GHQ-28, general health questionnaire-28;GS, Goldberg scale; HIT-6, headache impact test-6; HI, headache index; HQoLQ, health quality of life questionnaire; HSCL, Hopkins symptoms checklist-25;IES, impact of event scale; MA, meta-analysis; MPQ, McGill Pain Questionnaire; MVKS, Modified Von Korf scale; NDI, neck disability index; NHP, Nottingham health profile; NP, neck pain; NPAD, neck pain and disability scale; NPQ, Northwick park neck pain questionnaire; NPRS, numeric pain rating scale; NS, non-specific neck pain; PDI, pain disability index; POMS, profile of mood states; PSFS, patient specific functional scale; QDASH, quick disabilities of the arm, shoulder and hand;SF-12, 12 item short form questionnaire; SF-36, 36 item short form questionnaire; SF-MPQ, short form McGill pain questionnaire; SR, systematic review; TSK, Tampa scale of kinesiophobia; VAS, visual analog scale; VNPDI, Vernon neck pain disability index; WAD, whiplash associated disorder

**Table 2:** The top five patient-reported outcome measure stratified by construct ^a^

Rank ^b^	Disability (n = 13)	Pain Intensity (n = 4)	Psychosocial (n = 11)	QoL (n = 4)
1	NDI (33)	VAS (28)	FABQ (5)	SF-36 (11)
2	NPQ (13)	NPRS (27)	TSK (3)	SF-12 (4)
3	PSFS (5)	MPQ (2)	BDI (1)	EQ-5D (1)
4	NPAD (4)	SF-MPQ (1)	CES-D (1)	HQoLQ (1)
5	DASH (3)	*	GSES (1)	*

^a^ Total number of studies represented in parenthesis. BDI, Becks Depression Inventory; CES-D, Center for Epidemiologic Studies Depression Scale; DASH, Disabilities of the Arm, Shoulder and Hand; EQ-5D, EuroQoL-5D; FABQ, Fear Avoidance Belief Questionnaire; GSES, General Self-Efficacy Scale; HQoLQ, Health Quality of Life Questionnaire; MPQ, McGill Pain Questionnaire; NDI, Neck Disability Index; NPAD, Neck Pain and Disability Scale; NPQ, Northwick Park Neck Pain Questionnaire; NPRS, Numeric Pain Rating Scale; PSFS, Patient Specific Functional Scale; SF-12, 12 item Short Form Questionnaire; SF-36, 36 item Short Form Questionnaire; SF-MPQ, Short Form McGill Pain Questionnaire; TSK, Tampa Scale of Kinesiophobia; VAS, Visual Analog Scale

^b^ From highest frequency to lowest

* Did not have additional PROMs

### Patient-reported outcome measure constructs

#### Disability

**Fig. 2:**
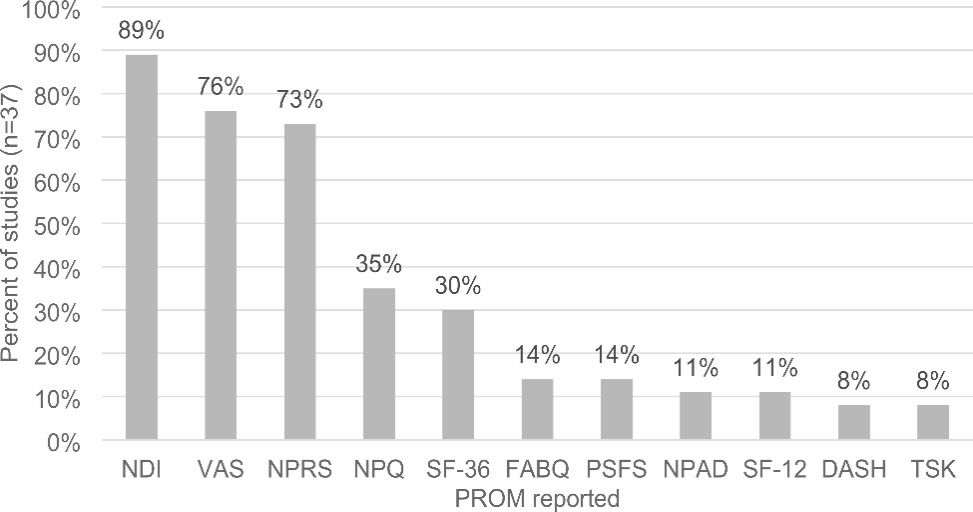
The 11 most frequently reported PROMs. *DASH, Disabilities of the Arm, Shoulder and Hand; FABQ, Fear Avoidance Belief Questionnaire; NDI, Neck Disability Index; NPAD, Neck Pain and Disability Scale; NPQ, Neck Pain Questionnaire; NPRS, Numeric Pain Rating Scale; PSFS, Patient Specific Functional Scale; SF-12, Short Form Health Survey-12; SF-36, Short Form Health Survey-36; TSK, Tampa Scale of Kinesiophobia; VAS, Visual Analog Scale

Details of the PROMs included in three or more reviews are presented in Fig. 2. Of the eleven PROMs that were represented in three or more reviews, 45% (n = 5) of the PROMs assessed disability. The most frequently reported PROMs measuring disability included the Neck Disability Index (NDI), Neck Pain Questionnaire (NPQ), Patient Specific Functional Scale (PSFS), Neck Pain and Disability Scale (NPAD), and Disabilities of the arm, shoulder and hand (DASH). The NDI was represented in 89% (n = 33) of studies and was the most frequently included measure of disability within our review. This was followed by the NPQ, PSFS and DASH represented in 35%, 14% and 8% of studies respectively. The aforementioned PROMs context of use was for diagnosis and prognosis in patients with neck pain and cervicogenic headache.

#### Pain intensity

13% (n = 4) of all included PROMs (n = 31) measured the construct of pain intensity. Four measures of pain intensity were represented in this review and only two PROMs were represented in three or more reviews. These included the visual analog scale (VAS) and numeric pain rating scale (NPRS). The VAS was the most frequently utilized pain intensity measure and was represented in 76% (n = 28) of included studies. The NPRS was included in 73% (n = 27) of studies.

#### Psychosocial

Ten measures of psychosocial function were represented in the included studies, with two PROMs represented in three or more reviews. These included the Fear Avoidance Belief Questionnaire (FABQ) and Tampa Scale of Kinesiophobia (TSK). The FABQ was the most utilized of the two measures, representing 14% (n = 5) of all reviews. The second most frequently utilized measure of psychosocial function was the TSK, represented in 8% (n = 3) of all reviews.

#### Quality of life

Four QoL measures were represented in the included 31 PROMs with two PROMs present in three or more reviews. These included the Short Form Health Survey-12(SF-12) and Short Form Health Survey-36(SF-36). The SF-36 was the most frequently utilized QoL measure and was represented in 30% (n = 11) of included reviews. The SF-12 was the second most frequently utilized QoL measure and was included in 11% (n = 4) of reviews with risk of bias ratings of low (2) and high (2).

#### Risk of bias

**Fig. 3:**
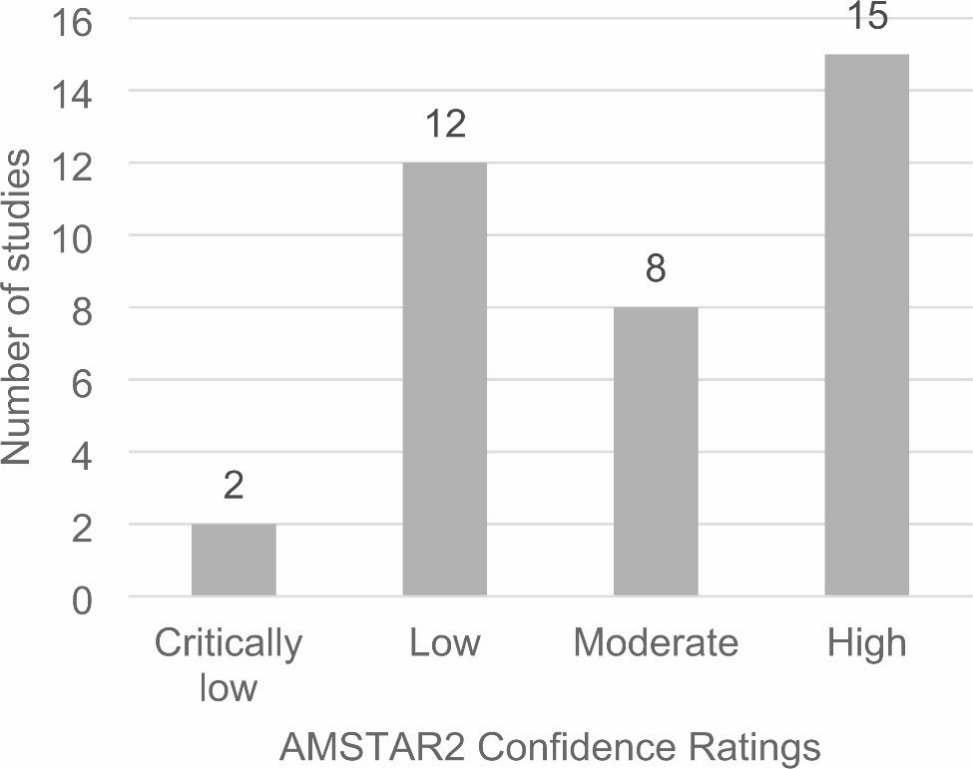
AMSTAR2(A MeaSurement Tool to Assess systematic Reviews) Confidence Ratings of included reviews

A summary of the results from the critical appraisal of 37 studies using the AMSTAR2 are described in Fig. 3 with full details provided in supplementary materials 1. Confidence in the results were rated as critically low [19, 20], low [21–32], moderate [12, 33–39], and high [40–54]. The methodological weaknesses in the critically low and low rated studies are considered critical domains by AMSTAR2. These included a failure to adequately investigate publication bias and its impact on the results (9 studies), a lack of consideration of risk of bias when interpreting the results of the review (5 studies), or insufficient justification for excluding individual studies (3 studies). Studies rated as moderate were lacking information in more than one of the non-critical domains. This included not performing study selection in duplicate (1 study), not performing data extraction in duplicate (8 studies), lack of reporting on sources of funding for the studies included in the review (14 studies), not assessing the potential impact of RoB in individual studies on the results of the meta-analysis or other evidence synthesis (3 studies), lack of satisfactory explanation for, and discussion of, any heterogeneity observed in the results of the review (9 studies), or failing to report any potential sources of conflict of interest, including funding (4 studies).

## Discussion

The purpose of this review was to identify PROMs that are reported in patients with neck pain receiving physical therapy interventions and to provide guidance for physical therapists and other practitioners on PROM selection for patients with neck pain. Similar to the findings described in the Academy of Orthopaedic Physical Therapy(AOPT) Neck Pain CPG revision, our review found that the NDI was the most commonly utilized PROM [4]. The NDI has demonstrated high-quality evidence of good to excellent internal consistency, moderate to excellent test-retest reliability, and moderate quality evidence of poor to moderate responsiveness in patients with neck disorders [55].

Second and third to the NDI in the frequency of use for evaluation of perceived disability, were the NPQ and PSFS respectively. These findings were not surprising as they were consistent with another systematic review [55] that found the NDI and NPQ to be the most frequently utilized PROMs in physical therapy practice for patients with neck disorders. Bobos et al. demonstrated the NPQ to be the second most frequently evaluated PROM assessing disability in individuals with neck pain and moderate quality evidence demonstrating good to excellent internal consistency and good test-retest reliability [55]. Although the PSFS was originally developed to be used across a variety of conditions, moderate quality evidence of high test-retest reliability (ICC = 0.82 for cervical radiculopathy) of the PSFS has been found in patients with neck disorders [55, 56].

For the construct of pain intensity, both the VAS and NPRS were reported in over three quarters of the studies in our review. Not surprisingly, an international survey of researchers determined the NPRS to be the most widely used measure in primary care for patients presenting with neck pain [57]. The NPRS has demonstrated high-to-moderate-quality evidence of moderate to strong (0.58 to 0.93) test-retest reliability with a moderate association of concurrent construct validity between the NDI and VAS of r = 0.36 to 0.69 in patients with neck pain [57].

An in-depth understanding of the outcomes associated with physical therapy interventions in the treatment of neck pain is critical to enhancing the quality and effectiveness of clinical practice. Although there is no substitute for clinical experience and an evidence-based objective examination, the knowledge gathered from understanding a patient’s health status as they perceive it is equally, if not more important to recognize.

It’s critical to note the use of these instruments in clinical care. Standardized PROMs are intended to improve patient-centered care, measure intervention effectiveness, inform clinical decision making, quality improvement initiatives, and enhance shared decision making between the patient and the clinician. A recent systematic review summarizing patients’ experiences and perspectives of PROMs in clinical care found that patients’ perceived benefits of PROMs included a sense of empowerment, providing information to inform clinical planning, assessment, diagnosis and individualized treatment. However they also noted some common barriers to engagement including the PROMs perceived relevance, utility of questions, understanding the measures purpose and concerns about how information is applied clinically [58]. In accordance with patients, most clinicians value PROMs as long as they can be useful during the decision-making process. Noted barriers to their use include not having the infrastructure in place for data collection and when collection of PROMs disrupts their normal workflow [59].

Our review has some noted strengths. First, our study was a review of reviews, resulting in our confidence in the results of our study. Moreover, our findings were consistent with what has been reported in the recent literature. Following Cochrane guidance, our study methodology was thorough and robust creating the platform to capture as many relevant reviews as possible that met our a priori defined inclusion and exclusion criteria. Additionally, this study only reviewed systematic reviews, therefore it is possible that other PROMs have been evaluated for patients with neck pain or cervicogenic headache which have not been previously included in a systematic review analysis. However, it would be anticipated that the most frequently utilized PROMs for patients with neck pain would have been included in the selected reviews.

Our review also has some noted limitations which are important to acknowledge. A limitation of our study is a bias in established measures being reported at higher rates. For example, the NDI and the VAS were initially published in 1991 and 1921 respectively [60, 61]. Comparatively speaking, other disability measures such as the NPQ and the NPAD were published in 1994 and 1999 respectively [62, 63]. For other domains, the SF-36, SF-12, TSK and FBQ were published between 1991–1995 [64–67]. Therefore, no outcome measures in our study that were included in three or more reviews have been published in the last 20 years.

Additionally, there were PROMs that were not reported in our review that have emerged recently. Patient-Reported Outcomes Measurement Information System (PROMIS) measures were not reported in any of the included studies in this review. The PROMIS PROMs are gaining increasing popularity in clinical practice and research due to their psychometric properties and their ability to compare patient health and treatment outcomes across the continuum of care. A recent systematic review by Young et al. found that the PROMIS-Physical Function(PROMIS-PF) and PROMIS-Pain interference(PROMIS-PI) demonstrate moderate to strong correlations with the NDI, VAS, and SF-12 [68]. Additionally, there is increasing interest in lifestyle behaviors related to neck pain [69–72]. PROMs related to lifestyle behaviors such as sleep, which has been shown to contribute to neck pain intensity and outcomes, were not found within our review [72].

There were several research gaps that were identified by our study that highlight key areas for future research. Although there was moderate consistency in the reporting of PROMs within the disability and pain intensity constructs, our review found much lower rates of reporting and higher variability within the psychosocial construct. Psychosocial measures represented 32% of all PROMs however these varied greatly (10 total) with 80% (8 out of 10) used in only one review across the 37 included studies. Given the prevalence of psychosocial factors that may influence neck pain intensity [73, 74],prognosis [4, 20, 74, 75], and treatment approaches [38, 76], these data suggest that psychosocial measures are infrequently and inconsistently used when evaluating patients with neck pain. Thus, it seems reasonable to suggest that a gap exists on which measures assessing psychosocial factors are psychometrically supported and valuable in clinical practice, therefore resulting in this mass heterogeneity. This finding suggests a need for future research and specific recommendations for psychosocial PROMs that may be used in clinical practice and research in patients with neck pain.

Another measure which was not found in our review, is the Optimal Screening for Prediction of Referral and Outcome Yellow Flag (OSPRO–YF) tool. Although psychological characteristics can present independently, for example as either depression or anxiety, in patients with chronic pain they often coexist [77]. The evaluation of multiple domains of psychological distress including depression, anxiety, and pain catastrophizing allows for increased effectiveness and efficiency in discriminating between patients who may be at risk for poor outcomes. This comprehensive evaluation also allows for classifying pain phenotypes and identifying those who would benefit from targeted treatment interventions such as cognitive behavioral therapy or psychologically informed treatment [77–79]. Due to this, considering a multidimensional tool which evaluates a global psychological profile would inform a clinician if specific targeted interventions would be beneficial for their patient. The OSPRO-YF tool was originally published in 2016 and combines 11 unidimensional psychological questionnaires into 3 domains (negative mood, fear avoidance and positive affect). This single questionnaire has been shown to have good accuracy estimating individual, full-length psychological questionnaire scores for depressive symptoms, anxiety, anger, fear-avoidance beliefs, kinesiophobia, catastrophizing, self-efficacy, and pain acceptance in those with neck pain [80–82].

In contrast to the findings within the psychosocial construct, our review found consistency of PROMs within the construct of QoL, with 41% of studies reporting the use of the SF-36 or the shorter version SF-12. However, both measures have associated costs, are lengthy and have high clinician and patient burden. Therefore clinically, we are not able to confidently recommend the SF-36 or 12 without taking these barriers into consideration and understanding contextual factors including the resources that are available to a clinic setting or clinician.

There are several key implications of our findings. First, our study highlights the need for minimal mandates of PROMs that capture the full spectrum of neck pain-related constructs, including psychosocial factors. This has important implications for both clinical practice and research, as a comprehensive understanding of patients’ health as they perceive it is crucial for providing optimal care, facilitating shared-decision making, measuring intervention effectiveness, informing clinical decision making and high quality research. Second, our review identified a core set of patient-reported outcome measures that demonstrate clinical value to clinicians and patients. We provide recommendations of these measures in clinical practice and research settings, aiming to improve the standardization and comparability of patient-reported outcomes across studies and interventions.

## Conclusion

There is great variability in PROMs used for patients with neck pain in physical therapy research and clinical practice. Based on these findings, we suggest that future research for neck pain evaluate PROMs that are most reported and psychometrically supported in the literature while considering clinician and patient burden. Based on the findings from this review, in the context of other literature, we recommend a core set of PROMs evaluating disability and pain intensity. This includes the NDI and NPRS or VAS. Assessment of patient QoL is critical, however recommendations for QoL PROMs need to be considered in the context of available resources and administrative burden. The findings from this review provides empirical evidence to assist in informing clinicians and researchers on the use of patient-reported outcome measures for patients with neck pain seeking physical therapy. Further research is needed to confidently recommend a QoL and psychosocial measure for patients presenting with neck pain. Other measures that were not included in this review but should be further evaluated for patients with neck pain are the PROMIS-PF, PROMIS-PI and the OSPRO-YF tool.

### Electronic supplementary material

Below is the link to the electronic supplementary material.

Supplementary Material 1

Supplementary Material 2

## Data Availability

All data generated or analyzed during this study are included in this published article and its additional files.

## Abbreviations

ADLQ: Activities of daily living questionnaire
AMSTAR2: Assessment of Multiple Systematic Reviews 2
AOPT: Academy of Orthopaedic Physical Therapy
BDI: Becks depression inventory
CADTH: Canadian Agency for Drugs and Technologies in Health
CES-D: Center for epidemiologic studies depression scale
CPG: Clinical Practice Guideline
COSMIN: COnsensus-based Standards for the selection of health status measurement Instruments
DASH: Disabilities of the arm, shoulder and hand
EQ-5D: EuroQoL-5D
FABQ: Fear avoidance belief questionnaire
GSES: General self-efficacy scale
GHQ-28: General health questionnaire-28
GS: Goldberg scale
HIT-6: Headache impact test-6
HI: Headache index
HqoLQ: Health quality of life questionnaire
HSCL: Hopkins symptoms checklist-25
IES: Impact of event scale
MPQ: McGill Pain Questionnaire
MVKS: Modified Von Korf scale
NDI: Neck disability index
NHP: Nottingham health profile
NPAD: Neck pain and disability scale
NPQ: Northwick park neck pain questionnaire
NPRS: Numeric pain rating scale
OSPRO-YF: Optimal Screening for Prediction of Referral and Outcome-Yellow Flag
PDI: Pain disability index
PICO: Population, Intervention, Comparison, Outcome
POMS: Profile of mood states
PRESS: Peer Review of Electronic Search Strategies
PRISMA: Preferred Reporting Items for Systematic Reviews and Meta-analysis
PROMIS: Patient-Reported Outcomes Measurement Information System
PROMIS-PF: Patient-Reported Outcomes Measurement Information System- Physical Function
PROMIS-PI: Patient-Reported Outcomes Measurement Information System- Pain interference
PROMs: Patient-reported outcome measures
PROSPERO: Prospective Register of Systematic Reviews
PSFS: Patient specific functional scale
QDASH: Quick disabilities of the arm, shoulder and hand
SF-12: 12 item short form questionnaire
SF-36: 36 item short form questionnaire
SF-MPQ: Short form McGill pain questionnaire
TSK: Tampa scale of kinesiophobia
QoL: Quality of life
VAS: Visual analog scale
VNPDI: Vernon neck pain disability index
WAD: Whiplash-associated disorder
