# Supplementary material for: Patient-reported outcome measures in physical therapy practice for neck pain: an overview of reviews
Source: J Patient Rep Outcomes. 2023 Oct 2;7:97. doi: 10.1186/s41687-023-00637-0 (PMC10545655; doi:10.1186/s41687-023-00637-0)
Supplement: Supplementary file 1 — Supplementary Material 1 [file 41687_2023_637_MOESM1_ESM.docx]

**Supplementary Material**

**Supplementary Table 1**. AMSTAR2^†^ details of overall confidence rating for included reviews.

| Study | 1 | 2 | 3 | 4 | 5 | 6 | 7 | 8 | 9 | 10 | 11 | 12 | 13 | 14 | 15 | 16 | Overall rating |
| --- | --- | --- | --- | --- | --- | --- | --- | --- | --- | --- | --- | --- | --- | --- | --- | --- | --- |
| Amiri et al.[[33]](https://paperpile.com/c/Yu678l/qhlh) | Y | PY | Y | Y | Y | N | PY | PY | Y | Y | N/A | N/A | Y | N | N/A | Y | Moderate |
| Araujo et al.[[34]](https://paperpile.com/c/Yu678l/UIBl) | Y | Y | Y | PY | Y | Y | Y | PY | Y | N | Y | Y | Y | Y | Y | N | Moderate |
| Borrella-Andrés et al[[21]](https://paperpile.com/c/Yu678l/PIui) | Y | PY | Y | PY | Y | N | Y | Y | Y | N | N/A | N/A | N | N | N/A | Y | Low |
| Chaibi A et al.[[54]](https://paperpile.com/c/Yu678l/pSXa) | Y | Y | Y | Y | Y | Y | Y | PY | Y | Y | Y | Y | Y | Y | Y | Y | High |
| Cox L et al.[[12]](https://paperpile.com/c/Yu678l/s3az) | Y | Y | Y | Y | Y | N | Y | PY | Y | Y | Y | N | Y | Y | Y | Y | Moderate |
| Dorji K et al.[[35]](https://paperpile.com/c/Yu678l/wMlP) | Y | PY | Y | PY | Y | Y | Y | Y | Y | Y | N/A | N/A | Y | N | N/A | Y | Moderate |
| Fernandez M et al[[32]](https://paperpile.com/c/Yu678l/hgmG) | Y | Y | Y | Y | Y | Y | Y | PY | Y | N | Y | N | N | Y | Y | Y | Low |
| Fredin K. et al.[[22]](https://paperpile.com/c/Yu678l/QPo6) | Y | Y | Y | PY | Y | Y | Y | Y | Y | Y | Y | Y | Y | N | N | Y | Low |
| Garzonio S et al.[[23]](https://paperpile.com/c/Yu678l/Acuy) | Y | Y | Y | Y | Y | Y | Y | Y | Y | N | Y | Y | Y | Y | N | Y | Low |
| Gross A et al.[[40]](https://paperpile.com/c/Yu678l/bNZZ) | Y | Y | Y | Y | Y | Y | Y | Y | Y | Y | N/A | N/A | Y | Y | N/A | Y | High |
| Hanel J et al.[[41]](https://paperpile.com/c/Yu678l/pFKT) | Y | Y | Y | PY | Y | Y | Y | Y | Y | Y | Y | Y | Y | N | Y | Y | High |
| Lantz JM et al.[[36]](https://paperpile.com/c/Yu678l/IGgC) | Y | PY | N | PY | Y | Y | Y | Y | PY | N | N/A | N/A | Y | N | N/A | Y | Moderate |
| Liang L et al.[[24]](https://paperpile.com/c/Yu678l/vkDi) | Y | Y | Y | PY | Y | Y | N | PY | Y | N | Y | Y | Y | Y | Y | Y | Low |
| Lin KY et al.[[25]](https://paperpile.com/c/Yu678l/jPKb) | Y | Y | Y | PY | Y | Y | Y | PY | Y | Y | Y | Y | Y | N | N | Y | Low |
| Louw S et al.[[26]](https://paperpile.com/c/Yu678l/ceEZ) | Y | PY | Y | PY | Y | Y | PY | Y | Y | N | Y | Y | Y | Y | N | Y | Low |
| Mallard F et al.[[42]](https://paperpile.com/c/Yu678l/jrRi) | Y | Y | Y | Y | Y | Y | Y | Y | Y | Y | N/A | N/A | Y | Y | N/A | Y | High |
| Martimbianco A et al.[[43]](https://paperpile.com/c/Yu678l/VRI4) | Y | Y | Y | Y | Y | Y | Y | Y | Y | Y | N/A | N/A | Y | Y | N/A | Y | High |
| Martin-Gomez C. et al.[[44]](https://paperpile.com/c/Yu678l/Rijx) | Y | Y | Y | Y | Y | Y | Y | Y | Y | Y | Y | Y | Y | Y | Y | Y | High |
| Masaracchio M et al.[[45]](https://paperpile.com/c/Yu678l/N67d) | Y | Y | Y | Y | Y | N | Y | Y | Y | Y | Y | Y | Y | Y | Y | Y | High |
| Monticone M. et al.[[27]](https://paperpile.com/c/Yu678l/LJ1T) | Y | Y | Y | Y | Y | Y | Y | Y | Y | N | Y | Y | Y | Y | N | Y | Low |
| Nunez Cabaleiro et al.[[28]](https://paperpile.com/c/Yu678l/95oX) | Y | Y | Y | Y | Y | Y | Y | Y | PY | N | N/A | N/A | N | N | N/A | Y | Low |
| Price J et al.[[46]](https://paperpile.com/c/Yu678l/SFiE) | Y | Y | Y | Y | Y | Y | Y | Y | Y | Y | N/A | N/A | Y | Y | N/A | Y | High |
| Qing W. et al.[[37]](https://paperpile.com/c/Yu678l/JxLR) | Y | Y | Y | PY | Y | Y | Y | Y | PY | N | Y | Y | Y | Y | Y | N | Moderate |
| Rampazo E. et al.[[47]](https://paperpile.com/c/Yu678l/HI5F) | Y | Y | Y | Y | Y | N | Y | Y | Y | Y | Y | Y | Y | Y | Y | Y | High |
| Rodriguez-Huguet et al[[39]](https://paperpile.com/c/Yu678l/oQ7V) | Y | Y | Y | Y | Y | N | Y | Y | Y | Y | N/A | N/A | Y | Y | N/A | Y | Moderate |
| Romeo A. et al.[[29]](https://paperpile.com/c/Yu678l/eQak) | Y | Y | Y | Y | Y | Y | Y | Y | Y | N | Y | Y | Y | Y | N | Y | Low |
| Southerst D. et al.[[38]](https://paperpile.com/c/Yu678l/Nhvc) | Y | Y | Y | Y | Y | Y | PY | Y | Y | Y | N/A | N/A | Y | Y | N/A | Y | Moderate |
| Tsiringakis G et al.[[48]](https://paperpile.com/c/Yu678l/Gub8) | Y | Y | Y | Y | Y | Y | Y | Y | Y | Y | Y | Y | Y | Y | Y | Y | High |
| Varangot-Reille et al[[49]](https://paperpile.com/c/Yu678l/9jfe) | Y | Y | Y | Y | Y | Y | Y | Y | Y | Y | Y | Y | Y | Y | Y | Y | High |
| Villanueva-Ruiz I et al[[30]](https://paperpile.com/c/Yu678l/65dH) | Y | Y | Y | Y | Y | Y | N | Y | Y | Y | Y | Y | Y | Y | Y | Y | Low |
| Visvanathan R et al[[19]](https://paperpile.com/c/Yu678l/giEY) | Y | N | Y | Y | N | N | N | PY | Y | N | N/A | N/A | N | N | N | Y | Critically low |
| Wang S et al.[[50]](https://paperpile.com/c/Yu678l/pgH3) | Y | Y | Y | Y | Y | Y | Y | PY | Y | N | Y | Y | Y | Y | Y | Y | High |
| Wilhelm M et al.[[51]](https://paperpile.com/c/Yu678l/SfaZ) | Y | Y | Y | PY | Y | Y | Y | Y | Y | Y | N/A | Y | Y | Y | Y | Y | High |
| Wu. et al[[83]](https://paperpile.com/c/Yu678l/LByB) | Y | Y | Y | Y | Y | Y | Y | PY | N | N | Y | N | N | Y | N | Y | Critically low |
| Yang J et al[[31]](https://paperpile.com/c/Yu678l/NHL1) | Y | PY | Y | Y | Y | Y | Y | Y | Y | Y | Y | Y | Y | Y | N | N | Low |
| Yu H. et al[[52]](https://paperpile.com/c/Yu678l/rpZS) | Y | Y | Y | Y | Y | N | Y | PY | Y | Y | N/A | N/A | Y | Y | N/A | Y | High |
| Zacharakis A et al[[53]](https://paperpile.com/c/Yu678l/Oc6D) | Y | Y | Y | Y | Y | Y | Y | Y | PY | Y | N/A | N/A | Y | Y | N/A | N | High |

Abbreviations: Y, yes; N, no; PY, partial yes; N/A, not applicable

^†^ 1. **Did the research questions and inclusion criteria for the review include the components of PICO? 2. Did the report of the review contain an explicit statement that the review methods were established prior to the conduct of the review and did the report justify any significant deviations from the protocol? 3. Did the review authors explain their selection of the study designs for inclusion in the review? 4. Did the review authors use a comprehensive literature search strategy? 5. Did the review authors perform study selection in duplicate? 6. Did the review authors perform data extraction in duplicate? 7. Did the review authors provide a list of excluded studies and justify the exclusions? 8. Did the review authors describe the included studies in adequate detail? 9. Did the review authors use a satisfactory technique for assessing the risk of bias (RoB) in individual studies that were included in the review? 10. Did the review authors report on the sources of funding for the studies included in the review? 11. If meta-analysis was performed did the review authors use appropriate methods for statistical combination of results? 12. If meta-analysis was performed, did the review authors assess the potential impact of RoB in individual studies on the results of the meta-analysis or other evidence synthesis? 13. Did the review authors account for RoB in individual studies when interpreting/ discussing the results of the review? 14. Did the review authors provide a satisfactory explanation for, and discussion of, any heterogeneity observed in the results of the review? 15. If they performed quantitative synthesis did the review authors carry out an adequate investigation of publication bias (small study bias) and discuss its likely impact on the results of the review? 16. Did the review authors report any potential sources of conflict of interest, including any funding they received for conducting the review?**

**Supplementary Table 2**. Search Strategy details

Total number of articles (before deduplication): 9457

Total number of articles (after deduplication): 7003

Database: MEDLINE (Ovid)

| 1.  Neck pain | Neck Pain/ OR exp Brachial Plexus Neuropathies/ OR exp neck injuries/ OR exp whiplash injuries/ OR thoracic outlet syndrome/ OR cervical rib syndrome/ OR Torticollis/ OR exp brachial plexus neuropathies/ OR exp brachial plexus neuritis/ OR myofascial pain syndromes/ OR exp Spinal Osteophytosis/ OR (neckache OR neckaches OR neckpain OR neckpains OR whiplash OR whiplashes OR whiplashing OR whiplashed OR cervicodynia? OR cervicalgia? OR brachialgia? OR torticollis OR "myofascial pain syndrome" OR "myofascial pain syndromes" OR "thoracic outlet syndrome" OR "thoracic outlet syndromes" OR "thorax outlet syndrome" OR "spinal osteophytosis").ti,ab. | | 34766 | |  |
| --- | --- | --- | --- | --- | --- |
| 2.  Neck | exp neck/ OR neck muscles/ OR exp cervical plexus/ OR atlanto-axial joint/ OR atlanto-occipital joint/ OR Cervical Atlas/ OR spinal nerve roots/ OR exp brachial plexus/ OR axis, cervical vertebra/ OR odontoid process/ OR Thoracic Vertebrae/ OR exp cervical vertebrae/ OR Intervertebral Disk/ OR (neck OR necks OR C1 OR C2 OR C3 OR C4 OR C5 OR C6 OR C7 OR T1 OR T2 OR T3 OR T4 OR T5 OR T6 OR T7 OR T8 OR T9 OR T10 OR T11 OR T12 OR odontoid OR odontoids OR occipital OR occipitalis OR occipitali?ation OR occipitali?ed OR occipito?cervical OR occipito?temporal OR atlanto OR atlanto?cervical OR atlanto?occipital OR atlanto?axial OR atlanto?dental OR brachial OR cervico?brachial OR cervico OR ((thoracic OR cervical) adj3 (vertebra OR vertebrae OR vertebraes OR vertebral OR spine OR spines OR spinal OR outlet OR outlets OR lumbar OR lumbral OR disk OR disks OR disc OR discs)) OR trapezius OR cervicogenic OR cervico?facial OR cervico?thoracic OR cervico?vertebral OR cervico?spinal OR cervical?brachial OR cranio?vertebral).ti,ab. | | 838830 | |  |
| 3.  exclude uterus terms | exp genital diseases, female/ OR exp *Uterus/ OR (genital disease* OR uterus OR uterine).ti,ab. | | 532865 | |  |
| 4. | 2 NOT 3 | | 831083 | |  |
| 5.  Pain or injuries with pain as a symptom | exp spinal diseases/ OR exp headache/ OR exp pain/ OR exp injuries/ OR exp "Sprains and Strains"/ OR Radiculopathy/ OR Polyradiculopathy/ OR exp Neuritis/ OR exp temporomandibular joint disorders/ OR exp temporomandibular joint dysfunction syndrome/ OR exp Arthritis/ OR Fibromyalgia/ OR spondylitis/ OR discitis/ OR spondylosis/ OR spondylolysis/ OR spondylolisthesis/ OR intervertebral disk degeneration/ OR intervertebral disk displacement/ OR (headache OR headaches OR pain OR pains OR pained OR painful* OR ache OR aches OR ached OR aching OR achy OR achiness sore OR soreness OR stiff OR stiffness OR discomfort OR discomforts OR uncomfortable* OR injury OR injuries OR injure OR injured OR injuring OR sprain OR sprains OR sprained OR strain OR strains OR strained OR monoradiculopathy OR monoradiculopathies OR mononeuropathy OR mononeuropathies OR radiculopathy OR radiculopathies OR polyradiculopathy OR polyradiculopathies OR neuritis OR neuropathy OR neuropathies OR neuropathic OR radiculitis OR (temporomandibular ADJ2 disorder?) OR arthriti? OR osteoarthriti? OR fibromyalgia? OR spondylosis OR spondylitis OR spondylolisthesis OR spondylosis OR spondylolysis OR spondylolyses OR spondylitis OR spondylolisthesis OR herniation? OR herniate OR herniates OR herniated OR herniating OR slipped OR prolapse OR prolapses OR prolapsed OR prolapsing OR displace OR displaces OR displaced OR displacement? OR bulg* OR osteophytosis OR discitis OR diskitis OR discopathy OR discopathies OR displacement? OR degeneration? OR degenerated OR degenerating OR neuralgia OR neuralgias).ti,ab. | | 4056019 | |  |
| 6. | 4 AND 5 | | 240777 | |  |
| 7. | 1 OR 6 | | 255391 | |  |
| 8.  Outcomes | exp treatment outcome/ OR exp treatment failure/ OR exp "Patient Reported Outcome Measures"/ OR Pain Measurement/ OR exp disability evaluation/ OR "Recovery of Function"/ OR exp "Activities of Daily Living"/ OR exp "Quality of Life"/ OR Questionnaires/ OR ("treatment outcome" OR "treatment outcomes" OR self?report OR self?reported OR self?reporting OR patient?reported OR patient?reporting OR patient?report OR patient?reports OR PROM OR PROMs OR PROMIS OR "PRO measure" OR "PRO measures" OR survival? OR response? OR impairment? OR impaired OR impairing OR disability OR disabilities OR disabled OR wellbeing OR well?being OR ((failure? OR failed OR termination? OR terminated OR terminate) AND (treatment? OR treated OR therap*)) OR ((physical* OR function*) ADJ2 (function* OR capacit* OR capabilit* OR limitation? OR performance*)) OR "activities of daily living" OR "activity of daily living" OR ADL OR ADLs OR IADL OR IADLs OR dexterity OR ambulat* OR "performance status" OR ((quality OR qualities) ADJ2 (life OR lives OR living OR lived)) OR HR-PRO OR HRPRO OR HRQL OR HRQoL OR QL OR QoL OR "health index*" OR "health indices" OR "health profile*" OR Toileting OR Bath OR Baths OR Bathe OR Bathes OR Bathed OR Bathing OR Wash OR Washes OR Washed OR Washing OR Shower OR Showers OR Showered OR Showering OR "personal hygiene" OR Dress OR Dresses OR Dressed OR Dressing OR Undress OR Undresses OR Undressed OR Undressing OR Walk OR Walks OR Walked OR Walking OR Stand OR Stands OR Stood OR Standing OR "Sit to stand" OR "changing position" OR "changing positions" OR "change position" OR "change positions" OR "maintain position" OR "maintain positions" OR "maintaining position" OR "maintaining positions" OR Kneel OR Kneels OR Kneeled OR Kneeling OR Bend OR Bends OR Bending OR Reach OR Reaching OR Eat OR Eating OR (climb* AND (stair OR stairs OR steps)) OR (toilet AND (use* OR usage)) OR (restroom AND (use* OR usage)) OR (participat* AND activit* AND (hobb* OR caregiv* OR volunteering OR gardening OR work OR job)) OR "complex activity" OR "complex activities").ti,ab. | 9998189 | | |  |
| 9. Methods, psychometric properties, validation, reproducibility, reliability | mt.fs. OR is.fs. OR (Validation Studies OR Comparative Study).pt. OR exp "Sensitivity and Specificity"/ OR factor analysis, statistical/ OR exp Outcome Assessment, Health Care/ OR exp Psychometrics/ OR exp Observer Variation/ OR exp Health Status Indicators/ OR exp "Reproducibility of Results"/ OR exp Discriminant Analysis/ OR (psychometr* OR outcome assessment OR observer variation OR reproducib* OR reliab* OR unreliab* OR valid* OR coefficient OR homogeneity OR homogeneous OR "internal consistency" OR (cronbach* AND (alpha OR alphas)) OR (item AND (correlation* OR selection* OR reduction*)) OR (agreement OR precision OR imprecision OR "precise values" OR test-retest) OR (test and retest) OR (reliab* AND (test OR retest)) OR stability OR interrater OR inter-rater OR intrarater OR intra-rater OR intertester OR inter-tester OR intratester OR intra-tester OR interobserver OR inter-observer OR intraobserver OR intraobserver or intertechnician OR inter-technician OR intratechnician OR intra-technician OR interexaminer OR inter-examiner OR intraexaminer OR intra-examiner OR interassay OR interassay OR intraassay OR intra-assay OR interindividual OR inter-individual OR intraindividual OR intra-individual OR interparticipant OR inter-participant OR intraparticipant OR intra-participant OR kappa OR kappas OR repeatab* OR ((replicab* OR repeated) AND (measure* OR findings OR result OR results OR test OR tests)) OR (generaliza* OR generalisa* or concordance) OR (intraclass AND correlation*) OR (discriminative OR "known group" OR factor analysis OR factor analyses OR dimension* OR subscale*) OR (multitrait AND scaling AND (analysis OR analyses)) OR (item discriminant OR interscale correlation* OR error OR errors OR "individual variability") OR (variability AND (analysis OR values)) OR (uncertainty AND (measurement OR measuring)) OR ("standard error of measurement" OR sensitiv* OR responsive*) OR ((minimal OR minimally OR clinical OR clinically) AND (important OR significant OR detectable) AND (change OR difference)) OR (small* AND (real OR detectable) AND (change OR difference)) OR (meaningful change OR "ceiling effect" OR "floor effect" OR "Item response model" OR IRT OR Rasch OR "Differential item functioning" OR DIF OR "computer adaptive testing" OR "item bank" OR "cross-cultural equivalence")).ti,ab. OR (clinimetr* OR clinometr* OR outcome measure*).tw. | 10941392 | | |  |
| 10. | 7 AND 8 AND 9 | | 64776 | |  |
| 11. | (systematic review or meta-analysis).pt. OR meta-analysis/ or systematic review/ or systematic reviews as topic/ or meta-analysis as topic/ or "meta analysis (topic)"/ or "systematic review (topic)"/ or network meta-analysis/ OR ((systematic* adj3 (review* or overview*)) or (methodologic* adj3 (review* or overview*))).ti,ab,kf,kw. OR ((quantitative adj3 (review* or overview* or synthes*)) or (research adj3 (integrati* or overview*))).ti,ab,kf,kw. OR ((integrative adj3 (review* or overview*)) or (collaborative adj3 (review* or overview*)) or (pool* adj3 analy*)).ti,ab,kf,kw. OR (data synthes* or data extraction* or data abstraction*).ti,ab,kf,kw. OR (handsearch* or hand search*).ti,ab,kf,kw. OR (mantel haenszel or peto or der simonian or dersimonian or fixed effect* or latin square*).ti,ab,kf,kw. OR (met analy* or metanaly*).ti,ab,kf,kw. OR (meta regression* or metaregression*).ti,ab,kf,kw. OR (meta-analy* or metaanaly* or systematic review*).mp,hw. OR (medline or cochrane or pubmed or medlars or embase or cinahl).ti,ab,hw. OR (cochrane or (health adj2 technology assessment) or evidence report).jw. OR (comparative adj3 (efficacy or effectiveness)).ti,ab,kf,kw. OR (outcomes research or relative effectiveness).ti,ab,kf,kw. OR ((indirect or indirect treatment or mixed-treatment or bayesian) adj3 comparison*).ti,ab,kf,kw. OR (multi* adj3 treatment adj3 comparison*).ti,ab,kf,kw. OR (mixed adj3 treatment adj3 (meta-analy* or metaanaly*)).ti,ab,kf,kw. OR umbrella review*.ti,ab,kf,kw. OR (multi* adj2 paramet* adj2 evidence adj2 synthesis).ti,ab,kw,kf. OR (multiparamet* adj2 evidence adj2 synthesis).ti,ab,kw,kf. OR (multi-paramet* adj2 evidence adj2 synthesis).ti,ab,kw,kf. OR exp clinical pathway/ OR exp clinical protocol/ OR clinical protocols/ OR exp consensus/ OR exp consensus development conference/ OR exp consensus development conferences as topic/ OR critical pathways/ OR exp guideline/ OR guidelines as topic/ OR exp practice guideline/ OR practice guidelines as topic/ OR health planning guidelines/ OR Clinical Decision Rules/ OR (guideline or practice guideline or consensus development conference or consensus development conference, NIH).pt. OR (position statement* or policy statement* or practice parameter* or best practice*).ti,ab,kf,kw. OR (standards or guideline or guidelines).ti,kf,kw. OR ((practice or treatment* or clinical) adj guideline*).ab. OR (CPG or CPGs).ti. OR consensus*.ti,kf,kw. OR consensus*.ab. /freq=2 OR ((critical or clinical or practice) adj2 (path or paths or pathway or pathways or protocol*)).ti,ab,kf,kw. OR recommendat*.ti,kf,kw. or guideline recommendation*.ab. OR (care adj2 (standard or path or paths or pathway or pathways or map or maps or plan or plans)).ti,ab,kf,kw. OR (algorithm* adj2 (screening or examination or test or tested or testing or assessment* or diagnosis or diagnoses or diagnosed or diagnosing)).ti,ab,kf,kw. OR (algorithm* adj2 (pharmacotherap* or chemotherap* or chemotreatment* or therap* or treatment* or intervention*)).ti,ab,kf,kw. OR (guideline* or standards or consensus* or recommendat*).au. OR (guideline* or standards or consensus* or recommendat*).ca. | | 1283570 | |  |
| 12. | 10 AND 11 | | 3856 | |  |
| 13. | Date Filter: 2016 to present | | 1826 | |  |
|  |  | |  |  | |

**Database: Embase (Elsevier)**

| 1. | 'neck pain'/exp OR 'brachial plexus neuropathy'/exp OR 'neck injury'/exp OR 'thorax outlet syndrome'/exp OR 'thorax outlet syndrome'/exp OR 'torticollis'/exp OR 'brachial plexus neuropathy'/exp OR 'brachial plexus neuropathy'/exp OR 'myofascial pain'/exp OR 'spondylosis'/exp OR (neckache OR neckaches OR neckpain OR neckpains OR whiplash OR whiplashes OR whiplashing OR whiplashed OR cervicodynia$ OR cervicalgia$ OR brachialgia$ OR torticollis OR 'myofascial pain syndrome' OR 'myofascial pain syndromes' OR 'thoracic outlet syndrome' OR 'thoracic outlet syndromes' OR 'thorax outlet syndrome' OR 'spinal osteophytosis'):ti,ab | 76552 |
| --- | --- | --- |
| 2. | 'neck'/exp OR 'neck muscle'/exp OR 'cervical plexus'/exp OR 'spinal nerve'/exp OR 'atlantoaxial joint'/exp OR 'atlantooccipital joint'/exp OR 'first cervical vertebra'/exp OR 'brachial plexus'/exp OR 'second cervical vertebra'/exp OR 'odontoid process'/exp OR 'thoracic spine'/exp OR 'cervical spine'/exp OR 'intervertebral disk'/exp OR (neck OR necks OR C1 OR C2 OR C3 OR C4 OR C5 OR C6 OR C7 OR T1 OR T2 OR T3 OR T4 OR T5 OR T6 OR T7 OR T8 OR T9 OR T10 OR T11 OR T12 OR odontoid OR odontoids OR occipital OR occipitalis OR occipitali$ation OR occipitali$ed OR occipito$cervical OR occipito$temporal OR atlanto OR atlanto$cervical OR atlanto$occipital OR atlanto$axial OR atlanto$dental OR brachial OR cervico$brachial OR cervico OR ((thoracic OR cervical) NEAR/3 (vertebra OR vertebrae OR vertebraes OR vertebral OR spine OR spines OR spinal OR outlet OR outlets OR lumbar OR lumbral OR disk OR disks OR disc OR discs)) OR trapezius OR cervicogenic OR cervico$facial OR cervico$thoracic OR cervico$vertebral OR cervico$spinal OR cervical$brachial OR cranio$vertebral):ti,ab | 1253887 |
| 3. | 'gynecologic disease'/exp OR 'uterus'/exp OR (genital disease* OR uterus OR uterine):ti,ab | 1002089 |
| 4. | 2 NOT 3 | 1229117 |
| 5. | 'spine disease'/exp OR (headache OR headaches OR pain OR pains OR pained OR painful* OR ache OR aches OR ached OR aching OR achy OR achiness sore OR soreness OR stiff OR stiffness OR discomfort OR discomforts OR uncomfortable* OR injury OR injuries OR injure OR injured OR injuring OR sprain OR sprains OR sprained OR strain OR strains OR strained OR monoradiculopathy OR monoradiculopathies OR mononeuropathy OR mononeuropathies OR radiculopathy OR radiculopathies OR polyradiculopathy OR polyradiculopathies OR neuritis OR neuropathy OR neuropathies OR neuropathic OR radiculitis OR (temporomandibular NEAR/2 disorder$) OR arthriti$ OR osteoarthriti$ OR fibromyalgia$ OR spondylosis OR spondylitis OR spondylolisthesis OR spondylosis OR spondylolysis OR spondylolyses OR spondylitis OR spondylolisthesis OR herniation$ OR herniate OR herniates OR herniated OR herniating OR slipped OR prolapse OR prolapses OR prolapsed OR prolapsing OR displace OR displaces OR displaced OR displacement$ OR bulg* OR osteophytosis OR discitis OR diskitis OR discopathy OR discopathies OR displacement$ OR degeneration$ OR degenerated OR degenerating OR neuralgia OR neuralgias):ti,ab | 3612274 |
| 6. | 4 AND 5 | 274973 |
| 7. | 1 OR 6 | 328692 |
| 8. | 'treatment outcome'/exp OR 'treatment failure'/exp OR 'patient-reported outcome'/exp OR 'pain measurement'/exp OR 'disability'/exp OR 'convalescence'/exp OR 'daily life activity'/exp OR 'quality of life'/exp OR 'questionnaire'/exp OR ('treatment outcome' OR 'treatment outcomes' OR self$report OR self$reported OR self$reporting OR patient$reported OR patient$reporting OR patient$report OR patient$reports OR PROM OR PROMs OR PROMIS OR 'PRO measure' OR 'PRO measures' OR survival$ OR response$ OR impairment$ OR impaired OR impairing OR disability OR disabilities OR disabled OR wellbeing OR well$being OR ((failure$ OR failed OR termination$ OR terminated OR terminate) AND (treatment$ OR treated OR therap*)) OR ((physical* OR function*) NEAR/2 (function* OR capacit* OR capabilit* OR limitation$ OR performance*)) OR 'activities of daily living' OR 'activity of daily living' OR ADL OR ADLs OR IADL OR IADLs OR dexterity OR ambulat* OR 'performance status' OR ((quality OR qualities) NEAR/2 (life OR lives OR living OR lived)) OR HR-PRO or HRPRO or HRQL or HRQoL or QL or QoL OR 'health index*' OR 'health indices' OR 'health profile*' OR Toileting OR Bath OR Baths OR Bathe OR Bathes OR Bathed OR Bathing OR Wash OR Washes OR Washed OR Washing OR Shower OR Showers OR Showered OR Showering OR 'Personal hygiene' OR Dress OR Dresses OR Dressed OR Dressing OR Undress OR Undresses OR Undressed OR Undressing OR Walk OR Walks OR Walked OR Walking OR Stand OR Stands OR Stood OR Standing OR 'Sit to stand' OR 'changing position' OR 'changing positions' OR 'change position' OR 'change positions' OR 'maintain position' OR 'maintain positions' OR 'maintaining position' OR 'maintaining positions' OR Kneel OR Kneels OR Kneeled OR Kneeling OR Bend OR Bends OR Bending OR Reach OR Reaching OR Eat OR Eating OR (climb* AND (stair OR stairs OR steps)) OR (Toilet AND (use OR usage)) OR (restroom AND (use OR usage)) OR (participat* AND activit* AND (hobb* OR caregiv* OR volunteering OR gardening OR work OR job)) OR 'complex activity' OR 'complex activities'):ti,ab | 13763444 |
| 9. | 'sensitivity and specificity'/exp OR 'factor analysis'/exp OR 'outcome assessment'/exp OR 'health care'/exp OR 'psychometry'/exp OR 'observer variation'/exp OR 'health status indicator'/exp OR 'reproducibility'/exp OR 'discriminant analysis'/exp OR (clinimetr* OR clinometr* OR outcome measure* OR psychometr* OR outcome assessment OR observer variation OR reproducib* OR reliab* OR unreliab* OR valid* OR coefficient OR homogeneity OR homogeneous OR 'internal consistency' OR (cronbach* AND (alpha OR alphas)) OR (item AND (correlation* OR selection* OR reduction*)) OR (agreement OR precision OR imprecision OR 'precise values' OR test-retest) OR (test and retest) OR (reliab* AND (test OR retest)) OR stability OR interrater OR inter-rater OR intrarater OR intra-rater OR intertester OR inter-tester OR intratester OR intra-tester OR interobserver OR inter-observer OR intraobserver OR intraobserver or intertechnician OR inter-technician OR intratechnician OR intra-technician OR interexaminer OR inter-examiner OR intraexaminer OR intra-examiner OR interassay OR interassay OR intraassay OR intra-assay OR interindividual OR inter-individual OR intraindividual OR intra-individual OR interparticipant OR inter-participant OR intraparticipant OR intra-participant OR kappa OR kappas OR repeatab* OR ((replicab* OR repeated) AND (measure* OR findings OR result OR results OR test OR tests)) OR (generaliza* OR generalisa* or concordance) OR (intraclass AND correlation*) OR (discriminative OR 'known group' OR factor analysis OR factor analyses OR dimension* OR subscale*) OR (multitrait AND scaling AND (analysis OR analyses)) OR (item discriminant OR interscale correlation* OR error OR errors OR 'individual variability') OR (variability AND (analysis OR values)) OR (uncertainty AND (measurement OR measuring)) OR ('standard error of measurement' OR sensitiv* OR responsive*) OR ((minimal OR minimally OR clinical OR clinically) AND (important OR significant OR detectable) AND (change OR difference)) OR (small* AND (real OR detectable) AND (change OR difference)) OR (meaningful change OR 'ceiling effect' OR 'floor effect' OR 'Item response model' OR IRT OR Rasch OR 'Differential item functioning' OR DIF OR 'computer adaptive testing' OR 'item bank' OR 'cross-cultural equivalence')):ti,ab | 12788819 |
| 10. | 7 AND 8 AND 9 | 73441 |
| 11. | 'meta analysis'/exp OR 'systematic review'/exp OR 'meta analysis (topic)'/exp OR 'systematic review topic'/exp OR (systematic* NEAR/3 (review* OR overview*)):ti,ab OR (methodologic* NEAR/3 (review* OR overview*)):ti,ab OR (quantitative NEAR/3 (review* OR overview* OR synthes*)):ti,ab OR (research NEAR/3 (integrati* OR overview*)):ti,ab OR (integrative NEAR/3 (review* OR overview*)):ti,ab OR (collaborative NEAR/3 (review* OR overview*)):ti,ab OR (pool* NEAR/3 analy*):ti,ab OR (comparative NEAR/3 (efficacy or effectiveness)):ti,ab OR ('data synthes*' OR 'data extraction*' OR 'data abstraction*' OR handsearch* OR (hand AND search*) OR 'mantel haenszel' OR peto OR 'der simonian' OR dersimonian OR 'fixed effect*' OR 'latin square*' OR 'met analy*' OR metanaly* OR 'meta regression*' OR metaregression* OR meta-analy* OR metaanaly* OR 'systematic review' OR 'systematic reviews' OR medline OR cochrane OR pubmed OR medlars OR embase OR cinahl OR cochrane OR 'evidence report' OR meta-analysis OR 'outcomes research' OR 'relative effectiveness'):ti,ab OR 'clinical pathway'/exp OR 'consensus'/exp OR 'clinical protocol'/exp OR 'consensus development'/exp OR 'practice guideline'/exp OR 'guideline'/exp OR (position statement* OR policy statement* OR 'practice parameter*' or best practice* OR standards or guideline or guidelines OR ((practice or treatment* or clinical) NEAR/1 guideline*) OR (CPG or CPGs) OR consensus* OR consensus* OR ((critical or clinical or practice) NEAR/2 (path or paths or pathway or pathways or protocol*)) OR recommendat* OR (care NEAR/2 (standard or path or paths or pathway or pathways or map or maps or plan or plans)) OR (algorithm* NEAR/2 (screening or examination or test or tested or testing or assessment* or diagnosis or diagnoses or diagnosed or diagnosing)) OR (algorithm* NEAR/2 (pharmacotherap* or chemotherap* or chemotreatment* or therap* or treatment* or intervention*))):ti,ab | 2699485 |
| 12. | 10 AND 11 | 7573 |
| 13. | 12 AND [01-01-2016]/sd | 4481 |
| 14 | 13 NOT ([conference abstract]/lim OR 'conference abstract'/exp OR 'conference abstract'/it) | 3043 |

**Database: CINAHL Complete (EBSCOhost)**

| 1. | MH "Neck Pain" OR MH "Brachial Plexus Neuropathies+" OR MH "Neck Injuries+" OR MH "Whiplash Injuries" OR MH "Thoracic Outlet Syndrome" OR MH "Torticollis" OR MH "Brachial Plexus Neuritis" OR MH "Myofascial Pain Syndromes+" OR MH "Osteoarthritis, Spine+" OR MH "Spinal Osteophytosis" OR MH "Osteoarthritis, Cervical" OR TI(neckache OR neckaches OR neckpain OR neckpains OR whiplash OR whiplashes OR whiplashing OR whiplashed OR cervicodynia# OR cervicalgia# OR brachialgia# OR torticollis OR "myofascial pain syndrome" OR "myofascial pain syndromes" OR "thoracic outlet syndrome" OR "thoracic outlet syndromes" OR "thorax outlet syndrome" OR "spinal osteophytosis) OR AB(neckache OR neckaches OR neckpain OR neckpains OR whiplash OR whiplashes OR whiplashing OR whiplashed OR cervicodynia# OR cervicalgia# OR brachialgia# OR torticollis OR "myofascial pain syndrome" OR "myofascial pain syndromes" OR "thoracic outlet syndrome" OR "thoracic outlet syndromes" OR "thorax outlet syndrome" OR "spinal osteophytosis) | 17237 |
| --- | --- | --- |
| 2. | MH "Neck+" OR MH "Neck Muscles+" OR MH "Cervical Plexus+" OR MH "Atlanto-Axial Joint" OR MH "Atlanto-Occipital Joint" OR MH "Cervical Atlas" OR MH "Spinal Nerve Roots+" OR MH "Brachial Plexus+" OR MH "Cervical Vertebrae+" OR MH "Thoracic Vertebrae" OR MH "Cervical Vertebrae+" OR MH "Intervertebral Disk+" OR TI(neck OR necks OR C1 OR C2 OR C3 OR C4 OR C5 OR C6 OR C7 OR T1 OR T2 OR T3 OR T4 OR T5 OR T6 OR T7 OR T8 OR T9 OR T10 OR T11 OR T12 OR odontoid OR odontoids OR occipital OR occipitalis OR occipitali#ation OR occipitali#ed OR occipito#cervical OR occipito#temporal OR atlanto OR atlanto#cervical OR atlanto#occipital OR atlanto#axial OR atlanto#dental OR brachial OR cervico#brachial OR cervico OR ((thoracic OR cervical) N3 (vertebra OR vertebrae OR vertebraes OR vertebral OR spine OR spines OR spinal OR outlet OR outlets OR lumbar OR lumbral OR disk OR disks OR disc OR discs)) OR trapezius OR cervicogenic OR cervico#facial OR cervico#thoracic OR cervico#vertebral OR cervico#spinal OR cervical#brachial OR cranio#vertebral) OR AB(neck OR necks OR C1 OR C2 OR C3 OR C4 OR C5 OR C6 OR C7 OR T1 OR T2 OR T3 OR T4 OR T5 OR T6 OR T7 OR T8 OR T9 OR T10 OR T11 OR T12 OR odontoid OR odontoids OR occipital OR occipitalis OR occipitali#ation OR occipitali#ed OR occipito#cervical OR occipito#temporal OR atlanto OR atlanto#cervical OR atlanto#occipital OR atlanto#axial OR atlanto#dental OR brachial OR cervico#brachial OR cervico OR ((thoracic OR cervical) N3 (vertebra OR vertebrae OR vertebraes OR vertebral OR spine OR spines OR spinal OR outlet OR outlets OR lumbar OR lumbral OR disk OR disks OR disc OR discs)) OR trapezius OR cervicogenic OR cervico#facial OR cervico#thoracic OR cervico#vertebral OR cervico#spinal OR cervical#brachial OR cranio#vertebral) | 143671 |
| 3. | MH "Genital Diseases, Female+" OR MH "Uterus+" OR TI(genital disease* OR uterus OR uterine) OR AB(genital disease* OR uterus OR uterine) | 140257 |
| 4. | 2 NOT 3 | 142337 |
| 5. | MH "Spinal Diseases+" OR MH "Headache+" OR MH "Pain+" OR MH "Wounds and Injuries+" OR MH "Sprains and Strains+" OR MH "Radiculopathy" OR MH "Polyradiculopathy+" OR MH "Neuritis+" OR MH "Temporomandibular Joint Diseases+" OR MH "Temporomandibular Joint Syndrome" OR MH "Arthritis+" OR MH "Fibromyalgia" OR MH "Spondylosis+" OR MH "Discitis" OR MH "Spondylolisthesis" OR MH "Intervertebral Disk Displacement" OR TI (headache OR headaches OR pain OR pains OR pained OR painful* OR ache OR aches OR ached OR aching OR achy OR achiness sore OR soreness OR stiff OR stiffness OR discomfort OR discomforts OR uncomfortable* OR injury OR injuries OR injure OR injured OR injuring OR sprain OR sprains OR sprained OR strain OR strains OR strained OR monoradiculopathy OR monoradiculopathies OR mononeuropathy OR mononeuropathies OR radiculopathy OR radiculopathies OR polyradiculopathy OR polyradiculopathies OR neuritis OR neuropathy OR neuropathies OR neuropathic OR radiculitis OR (temporomandibular N2 disorder#) OR arthriti# OR osteoarthriti# OR fibromyalgia# OR spondylosis OR spondylitis OR spondylolisthesis OR spondylosis OR spondylolysis OR spondylolyses OR spondylitis OR spondylolisthesis OR herniation# OR herniate OR herniates OR herniated OR herniating OR slipped OR prolapse OR prolapses OR prolapsed OR prolapsing OR displace OR displaces OR displaced OR displacement# OR bulg* OR osteophytosis OR discitis OR diskitis OR discopathy OR discopathies OR displacement# OR degeneration# OR degenerated OR degenerating OR neuralgia OR neuralgias) OR AB(headache OR headaches OR pain OR pains OR pained OR painful* OR ache OR aches OR ached OR aching OR achy OR achiness sore OR soreness OR stiff OR stiffness OR discomfort OR discomforts OR uncomfortable* OR injury OR injuries OR injure OR injured OR injuring OR sprain OR sprains OR sprained OR strain OR strains OR strained OR monoradiculopathy OR monoradiculopathies OR mononeuropathy OR mononeuropathies OR radiculopathy OR radiculopathies OR polyradiculopathy OR polyradiculopathies OR neuritis OR neuropathy OR neuropathies OR neuropathic OR radiculitis OR (temporomandibular N2 disorder#) OR arthriti# OR osteoarthriti# OR fibromyalgia# OR spondylosis OR spondylitis OR spondylolisthesis OR spondylosis OR spondylolysis OR spondylolyses OR spondylitis OR spondylolisthesis OR herniation# OR herniate OR herniates OR herniated OR herniating OR slipped OR prolapse OR prolapses OR prolapsed OR prolapsing OR displace OR displaces OR displaced OR displacement# OR bulg* OR osteophytosis OR discitis OR diskitis OR discopathy OR discopathies OR displacement# OR degeneration# OR degenerated OR degenerating OR neuralgia OR neuralgias) | 1022240 |
| 6. | 4 AND 5 | 63347 |
| 7. | 1 OR 6 | 70839 |
| 8. | (MH "Patient-Reported Outcomes+") OR (MH "Outcome Assessment") OR (MH "Treatment Outcomes+") OR (MH "Pain Measurement") OR (MH "Severity of Disability") OR (MH "Disability Evaluation+") OR (MH "Disability Management") OR  (MH "Functional Assessment+") OR (MH "Activities of Daily Living+") OR TI ("treatment outcome" OR "treatment outcomes" OR self#report OR self#reported OR self#reporting OR patient#reported OR patient#reporting OR patient#report OR patient#reports OR PROM OR PROMs OR PROMIS OR "PRO measure" OR "PRO measures" OR survival# OR response# OR impairment# OR impaired OR impairing OR disability OR disabilities OR disabled OR wellbeing OR well#being OR mobility OR "activities of daily living" OR "activity of daily living" OR ADL OR ADLs OR IADL OR IADLs OR dexterity OR ambulat* OR "performance status" OR "HR-PRO" OR "HRPRO" OR "HRQL" OR "HRQoL" OR "QL" OR "QoL" OR "health index*" OR "health indices" OR "health profile*" OR Toileting OR Bath OR Baths OR Bathe OR Bathes OR Bathed OR Bathing OR Wash OR Washes OR Washed OR Washing OR Shower OR Showers OR Showered OR Showering OR “Personal hygiene” OR Dress OR Dresses OR Dressed OR Dressing OR Undress OR Undresses OR Undressed OR Undressing OR Walk OR Walks OR Walked OR Walking OR Stand OR Stands OR Stood OR Standing OR “Sit to stand” OR "changing position" OR "changing positions" OR "change position" OR "change positions" OR "maintain position" OR "maintain positions" OR "maintaining position" OR "maintaining positions" OR Kneel OR Kneels OR Kneeled OR Kneeling OR Bend OR Bends OR Bending OR Reach OR Reaching OR Eat OR Eating) OR TI((failure# OR failed OR termination# OR terminated OR terminate) AND (treatment# OR treated OR therap*)) OR TI((physical* OR function*) N2 (function* OR capacit* OR capabilit* OR limitation# OR performance*)) OR TI((quality OR qualities) N2 (life OR lives OR living OR lived)) OR TI(climb* AND (stair OR stairs OR steps)) OR TI(Toilet AND (use OR usage)) OR TI(restroom AND (use OR usage)) OR TI(participat* AND activit* AND (hobb* OR caregiv* OR volunteering OR gardening OR work OR job)) OR "complex activity" OR "complex activities") OR AB("treatment outcome" OR "treatment outcomes" OR self#report OR self#reported OR self#reporting OR patient#reported OR patient#reporting OR patient#report OR patient#reports OR PROM OR PROMs OR PROMIS OR "PRO measure" OR "PRO measures" OR survival# OR response# OR impairment# OR impaired OR impairing OR disability OR disabilities OR disabled OR wellbeing OR well#being OR mobility OR "activities of daily living" OR "activity of daily living" OR ADL OR ADLs OR IADL OR IADLs OR dexterity OR ambulat* OR "performance status" OR "HR-PRO" OR "HRPRO" OR "HRQL" OR "HRQoL" OR "QL" OR "QoL" OR "health index*" OR "health indices" OR "health profile*" OR Toileting OR Bath OR Baths OR Bathe OR Bathes OR Bathed OR Bathing OR Wash OR Washes OR Washed OR Washing OR Shower OR Showers OR Showered OR Showering OR “Personal hygiene” OR Dress OR Dresses OR Dressed OR Dressing OR Undress OR Undresses OR Undressed OR Undressing OR Walk OR Walks OR Walked OR Walking OR Stand OR Stands OR Stood OR Standing OR “Sit to stand” OR "changing position" OR "changing positions" OR "change position" OR "change positions" OR "maintain position" OR "maintain positions" OR "maintaining position" OR "maintaining positions" OR Kneel OR Kneels OR Kneeled OR Kneeling OR Bend OR Bends OR Bending OR Reach OR Reaching OR Eat OR Eating) OR AB((failure# OR failed OR termination# OR terminated OR terminate) AND (treatment# OR treated OR therap*)) OR AB((physical* OR function*) N2 (function* OR capacit* OR capabilit* OR limitation# OR performance*)) OR AB((quality OR qualities) N2 (life OR lives OR living OR lived)) OR AB(climb* AND (stair OR stairs OR steps)) OR AB(Toilet AND (use OR usage)) OR AB(restroom AND (use OR usage)) OR AB(participat* AND activit* AND (hobb* OR caregiv* OR volunteering OR gardening OR work OR job)) OR "complex activity" OR "complex activities") | 1711518 |
| 9. | MH "Sensitivity and Specificity" OR MH "Data Analysis, Statistical+" OR MH "Outcome Assessment" OR MH "Psychometrics" OR MH "Health Status Indicators" OR MH "Reproducibility of Results" OR MH "Discriminant Analysis" OR TI(psychometr* OR outcome assessment OR observer variation OR reproducib* OR reliab* OR unreliab* OR valid* OR coefficient OR homogeneity OR homogeneous OR "internal consistency" OR (cronbach* AND (alpha OR alphas)) OR (item AND (correlation* OR selection* OR reduction*)) OR (agreement OR precision OR imprecision OR "precise values" OR test-retest) OR (test and retest) OR (reliab* AND (test OR retest)) OR stability OR interrater OR inter-rater OR intrarater OR intra-rater OR intertester OR inter-tester OR intratester OR intra-tester OR interobserver OR inter-observer OR intraobserver OR intraobserver or intertechnician OR inter-technician OR intratechnician OR intra-technician OR interexaminer OR inter-examiner OR intraexaminer OR intra-examiner OR interassay OR interassay OR intraassay OR intra-assay OR interindividual OR inter-individual OR intraindividual OR intra-individual OR interparticipant OR inter-participant OR intraparticipant OR intra-participant OR kappa OR kappas OR repeatab* OR ((replicab* OR repeated) AND (measure* OR findings OR result OR results OR test OR tests)) OR (generaliza* OR generalisa* or concordance) OR (intraclass AND correlation*) OR (discriminative OR "known group" OR factor analysis OR factor analyses OR dimension* OR subscale*) OR (multitrait AND scaling AND (analysis OR analyses)) OR (item discriminant OR interscale correlation* OR error OR errors OR "individual variability") OR (variability AND (analysis OR values)) OR (uncertainty AND (measurement OR measuring)) OR ("standard error of measurement" OR sensitiv* OR responsive*) OR ((minimal OR minimally OR clinical OR clinically) AND (important OR significant OR detectable) AND (change OR difference)) OR (small* AND (real OR detectable) AND (change OR difference)) OR (meaningful change OR "ceiling effect" OR "floor effect" OR "Item response model" OR IRT OR Rasch OR "Differential item functioning" OR DIF OR "computer adaptive testing" OR "item bank" OR "cross-cultural equivalence") OR (clinimetr* OR clinometr* OR outcome measure*)) OR AB(psychometr* OR outcome assessment OR observer variation OR reproducib* OR reliab* OR unreliab* OR valid* OR coefficient OR homogeneity OR homogeneous OR "internal consistency" OR (cronbach* AND (alpha OR alphas)) OR (item AND (correlation* OR selection* OR reduction*)) OR (agreement OR precision OR imprecision OR "precise values" OR test-retest) OR (test and retest) OR (reliab* AND (test OR retest)) OR stability OR interrater OR inter-rater OR intrarater OR intra-rater OR intertester OR inter-tester OR intratester OR intra-tester OR interobserver OR inter-observer OR intraobserver OR intraobserver or intertechnician OR inter-technician OR intratechnician OR intra-technician OR interexaminer OR inter-examiner OR intraexaminer OR intra-examiner OR interassay OR interassay OR intraassay OR intra-assay OR interindividual OR inter-individual OR intraindividual OR intra-individual OR interparticipant OR inter-participant OR intraparticipant OR intra-participant OR kappa OR kappas OR repeatab* OR ((replicab* OR repeated) AND (measure* OR findings OR result OR results OR test OR tests)) OR (generaliza* OR generalisa* or concordance) OR (intraclass AND correlation*) OR (discriminative OR "known group" OR factor analysis OR factor analyses OR dimension* OR subscale*) OR (multitrait AND scaling AND (analysis OR analyses)) OR (item discriminant OR interscale correlation* OR error OR errors OR "individual variability") OR (variability AND (analysis OR values)) OR (uncertainty AND (measurement OR measuring)) OR ("standard error of measurement" OR sensitiv* OR responsive*) OR ((minimal OR minimally OR clinical OR clinically) AND (important OR significant OR detectable) AND (change OR difference)) OR (small* AND (real OR detectable) AND (change OR difference)) OR (meaningful change OR "ceiling effect" OR "floor effect" OR "Item response model" OR IRT OR Rasch OR "Differential item functioning" OR DIF OR "computer adaptive testing" OR "item bank" OR "cross-cultural equivalence") OR (clinimetr* OR clinometr* OR outcome measure*)) | 2008829 |
| 10. | 7 AND 8 AND 9 | 15443 |
| 11. | PT("Meta Analysis" OR "Meta Synthesis" OR "Systematic Review") OR MH "Meta Analysis" OR MH "Systematic Review" OR TI(((systematic* N3 (review* or overview*)) or (methodologic* N3 (review* or overview*))) OR ((quantitative N3 (review* or overview* or synthes*)) or (research N3 (integrati* or overview*))) OR ((integrative N3 (review* or overview*)) or (collaborative N3 (review* or overview*)) or (pool* N3 analy*)) OR (data synthes* or data extraction* or data abstraction*) OR (handsearch* or hand search*) OR (mantel haenszel or peto or der simonian or dersimonian or fixed effect* or latin square*) OR (met analy* or metanaly*) OR (meta regression* or metaregression*) OR (meta-analy* or metaanaly* or systematic review*) OR (medline or cochrane or pubmed or medlars or embase or cinahl OR cochrane) OR (meta-analysis or systematic review) OR (comparative N3 (efficacy or effectiveness)) OR (outcomes research or relative effectiveness) OR ((indirect or indirect treatment or mixed-treatment) N1 comparison*)) OR AB(((systematic* N3 (review* or overview*)) or (methodologic* N3 (review* or overview*))) OR ((quantitative N3 (review* or overview* or synthes*)) or (research N3 (integrati* or overview*))) OR ((integrative N3 (review* or overview*)) or (collaborative N3 (review* or overview*)) or (pool* N3 analy*)) OR (data synthes* or data extraction* or data abstraction*) OR (handsearch* or hand search*) OR (mantel haenszel or peto or der simonian or dersimonian or fixed effect* or latin square*) OR (met analy* or metanaly*) OR (meta regression* or metaregression*) OR (meta-analy* or metaanaly* or systematic review* or biomedical technology assessment* or bio-medical technology assessment*) OR (medline or cochrane or pubmed or medlars or embase or cinahl OR cochrane) OR (meta-analysis or systematic review) OR (comparative N3 (efficacy or effectiveness)) OR (outcomes research or relative effectiveness) OR ((indirect or indirect treatment or mixed-treatment) N1 comparison*)) OR PT("Practice Guidelines") OR MH "Critical Path" OR MH "Consensus" OR MH "Practice Guidelines" OR TI((position statement* or policy statement* or practice parameter* or best practice*) OR (standards or guideline or guidelines) OR ((practice or treatment* or clinical) N1 guideline*) OR (CPG or CPGs) OR consensus* OR consensus* OR ((critical or clinical or practice) N2 (path or paths or pathway or pathways or protocol*)) OR recommendat* OR (care N2 (standard or path or paths or pathway or pathways or map or maps or plan or plans)) OR (algorithm* N2 (screening or examination or test or tested or testing or assessment* or diagnosis or diagnoses or diagnosed or diagnosing)) OR (algorithm* N2 (pharmacotherap* or chemotherap* or chemotreatment* or therap* or treatment* or intervention*))) OR AB((position statement* or policy statement* or practice parameter* or best practice*) OR (standards or guideline or guidelines) OR ((practice or treatment* or clinical) N1 guideline*) OR (CPG or CPGs) OR consensus* OR consensus* OR ((critical or clinical or practice) N2 (path or paths or pathway or pathways or protocol*)) OR recommendat* OR (care N2 (standard or path or paths or pathway or pathways or map or maps or plan or plans)) OR (algorithm* N2 (screening or examination or test or tested or testing or assessment* or diagnosis or diagnoses or diagnosed or diagnosing)) OR (algorithm* N2 (pharmacotherap* or chemotherap* or chemotreatment* or therap* or treatment* or intervention*))) | 1014891 |
| 12. | 10 AND 11 | 3656 |
| 13. | date filter: 2016 - present | 1728 |

**Database: Web of Science (Clarivate)**

| 1.  Neck pain | TS=(neckache OR neckaches OR neckpain OR neckpains OR whiplash OR whiplashes OR whiplashing OR whiplashed OR cervicodynia? OR cervicalgia? OR brachialgia? OR torticollis OR "myofascial pain syndrome" OR "myofascial pain syndromes" OR "thoracic outlet syndrome" OR "thoracic outlet syndromes" OR "thorax outlet syndrome" OR "spinal osteophytosis") | 11634 |
| --- | --- | --- |
| 2.  Neck | TS=(neck OR necks OR C1 OR C2 OR C3 OR C4 OR C5 OR C6 OR C7 OR T1 OR T2 OR T3 OR T4 OR T5 OR T6 OR T7 OR T8 OR T9 OR T10 OR T11 OR T12 OR odontoid OR odontoids OR occipital OR occipitalis OR occipitali?ation OR occipitali?ed OR occipito?cervical OR occipito?temporal OR atlanto OR atlanto?cervical OR atlanto?occipital OR atlanto?axial OR atlanto?dental OR brachial OR cervico?brachial OR cervico OR ((thoracic OR cervical) near/3 (vertebra OR vertebrae OR vertebraes OR vertebral OR spine OR spines OR spinal OR outlet OR outlets OR lumbar OR lumbral OR disk OR disks OR disc OR discs)) OR trapezius OR cervicogenic OR cervico?facial OR cervico?thoracic OR cervico?vertebral OR cervico?spinal OR cervical?brachial OR cranio?vertebral) | 832775 |
| 3. | TS=( genital disease* OR uterus OR uterine) | 178622 |
| 4. | 2 NOT 3 | 829238 |
| 5. | TS=(headache OR headaches OR pain OR pains OR pained OR painful* OR ache OR aches OR ached OR aching OR achy OR achiness sore OR soreness OR stiff OR stiffness OR discomfort OR discomforts OR uncomfortable* OR injury OR injuries OR injure OR injured OR injuring OR sprain OR sprains OR sprained OR strain OR strains OR strained OR monoradiculopathy OR monoradiculopathies OR mononeuropathy OR mononeuropathies OR radiculopathy OR radiculopathies OR polyradiculopathy OR polyradiculopathies OR neuritis OR neuropathy OR neuropathies OR neuropathic OR radiculitis OR (temporomandibular NEAR/2 disorder?) OR arthriti? OR osteoarthriti? OR fibromyalgia? OR spondylosis OR spondylitis OR spondylolisthesis OR spondylosis OR spondylolysis OR spondylolyses OR spondylitis OR spondylolisthesis OR herniation? OR herniate OR herniates OR herniated OR herniating OR slipped OR prolapse OR prolapses OR prolapsed OR prolapsing OR displace OR displaces OR displaced OR displacement? OR bulg* OR osteophytosis OR discitis OR diskitis OR discopathy OR discopathies OR displacement? OR degeneration? OR degenerated OR degenerating OR neuralgia OR neuralgias) | 4437967 |
| 6. | 4 AND 5 | 164377 |
| 7. | 1 OR 6 | 171610 |
| 8. | TS=("treatment outcome" OR "treatment outcomes" OR self?report OR self?reported OR self?reporting OR patient?reported OR patient?reporting OR patient?report OR patient?reports OR PROM OR PROMs OR PROMIS OR "PRO measure" OR "PRO measures" OR survival? OR response? OR impairment? OR impaired OR impairing OR disability OR disabilities OR disabled OR wellbeing OR well?being OR mobility OR ((failure? OR failed OR termination? OR terminated OR terminate) AND (treatment? OR treated OR therap*)) OR ((physical* OR function*) near/2 (function* OR capacit* OR capabilit* OR limitation? OR performance*)) OR "activities of daily living" OR "activity of daily living" OR ADL OR ADLs OR IADL OR IADLs OR dexterity OR ambulat* OR "performance status" OR ((quality OR qualities) near/2 (life OR lives OR living OR lived)) OR HR-PRO OR HRPRO OR HRQL OR HRQoL OR QL OR QoL OR "health index*" OR "health indices" OR "health profile*" OR Toileting OR Bath OR Baths OR Bathe OR Bathes OR Bathed OR Bathing OR Wash OR Washes OR Washed OR Washing OR Shower OR Showers OR Showered OR Showering OR "personal hygiene" OR Dress OR Dresses OR Dressed OR Dressing OR Undress OR Undresses OR Undressed OR Undressing OR Walk OR Walks OR Walked OR Walking OR Stand OR Stands OR Stood OR Standing OR "Sit to stand" OR "changing position" OR "changing positions" OR "change position" OR "change positions" OR "maintain position" OR "maintain positions" OR "maintaining position" OR "maintaining positions" OR Kneel OR Kneels OR Kneeled OR Kneeling OR Bend OR Bends OR Bending OR Reach OR Reaching OR Eat OR Eating OR (climb* AND (stair OR stairs OR steps)) OR (toilet AND (use* OR usage)) OR (restroom AND (use* OR usage)) OR (participat* AND activit* AND (hobb* OR caregiv* OR volunteering OR gardening OR work OR job)) OR "complex activity" OR "complex activities") | 12666488 |
| 9. | TS=(clinimetr* OR clinometr* OR "outcome measure*" OR psychometr* OR outcome assessment OR observer variation OR reproducib* OR reliab* OR unreliab* OR valid* OR coefficient OR homogeneity OR homogeneous OR "internal consistency" OR (cronbach* AND (alpha OR alphas)) OR (item AND (correlation* OR selection* OR reduction*)) OR (agreement OR precision OR imprecision OR "precise values" OR test-retest) OR (test and retest) OR (reliab* AND (test OR retest)) OR stability OR interrater OR inter-rater OR intrarater OR intra-rater OR intertester OR inter-tester OR intratester OR intra-tester OR interobserver OR inter-observer OR intraobserver OR intraobserver or intertechnician OR inter-technician OR intratechnician OR intra-technician OR interexaminer OR inter-examiner OR intraexaminer OR intra-examiner OR interassay OR interassay OR intraassay OR intra-assay OR interindividual OR inter-individual OR intraindividual OR intra-individual OR interparticipant OR inter-participant OR intraparticipant OR intra-participant OR kappa OR kappas OR repeatab* OR ((replicab* OR repeated) AND (measure* OR findings OR result OR results OR test OR tests)) OR (generaliza* OR generalisa* or concordance) OR (intraclass AND correlation*) OR (discriminative OR "known group" OR factor analysis OR factor analyses OR dimension* OR subscale*) OR (multitrait AND scaling AND (analysis OR analyses)) OR (item discriminant OR interscale correlation* OR error OR errors OR "individual variability") OR (variability AND (analysis OR values)) OR (uncertainty AND (measurement OR measuring)) OR ("standard error of measurement" OR sensitiv* OR responsive*) OR ((minimal OR minimally OR clinical OR clinically) AND (important OR significant OR detectable) AND (change OR difference)) OR (small* AND (real OR detectable) AND (change OR difference)) OR (meaningful change OR "ceiling effect" OR "floor effect" OR "Item response model" OR IRT OR Rasch OR "Differential item functioning" OR DIF OR "computer adaptive testing" OR "item bank" OR "cross-cultural equivalence")) | 16241935 |
| 10. | 7 AND 8 AND 9 | 27585 |
| 11. | TS=("data synthes*" or "data extraction*" OR "data abstraction*" OR handsearch* OR "hand search*" OR "mantel haenszel" OR peto OR "der simonian" OR dersimonian OR "fixed effect*" OR "latin square*" OR "met analy*" OR metanaly* OR "meta regression*" OR metaregression* OR meta-analy* OR metaanaly* OR "systematic review*" OR medline OR cochrane OR pubmed OR medlars OR embase OR cinahl OR cochrane OR "outcomes research" OR "relative effectiveness" OR "position statement*" OR "policy statement*" OR "practice parameter*" OR "best practice*" OR standards OR guideline OR guidelines OR CPG OR CPGs OR consensus* OR consensus* OR recommendat*) OR TS=(systematic* near/3 (review* OR overview*)) OR TS=(quantitative near/3 (review* OR overview* OR synthes*)) OR TS=(research near/3 (integrati* OR overview*)) OR TS=(integrative near/3 (review* OR overview*)) OR TS=(collaborative near/3 (review* OR overview*)) OR TS=(pool* near/3 analy*) OR TS=("evidence report" OR meta-analysis OR "systematic review") OR TS=((practice or treatment* or clinical) near/1 guideline*) OR TS=((critical or clinical or practice) near/2 (path or paths or pathway or pathways or protocol*)) OR TS=(care near/2 (standard or path or paths or pathway or pathways or map or maps or plan or plans)) OR TS=(algorithm* near/2 (screening or examination or test or tested or testing or assessment* or diagnosis or diagnoses or diagnosed or diagnosing)) OR TS=(algorithm* near/2 (pharmacotherap* or chemotherap* or chemotreatment* or therap* or treatment* or intervention*)) | 4388963 |
| 12. | 10 AND 11 | 5096 |
| 13. | date filter: 2016 - present | 2860 |
